# Quantifying randomness in real networks

**DOI:** 10.1038/ncomms9627

**Published:** 2015-10-20

**Authors:** Chiara Orsini, Marija M. Dankulov, Pol Colomer-de-Simón, Almerima Jamakovic, Priya Mahadevan, Amin Vahdat, Kevin E. Bassler, Zoltán Toroczkai, Marián Boguñá, Guido Caldarelli, Santo Fortunato, Dmitri Krioukov

**Affiliations:** 1 CAIDA, University of California San Diego, San Diego, California 92093, USA; 2 Information Engineering Department, University of Pisa, Pisa 56122, Italy; 3 Scientific Computing Laboratory, Institute of Physics Belgrade, University of Belgrade, Belgrade 11080, Serbia; 4 Department of Biomedical Engineering and Computational Science, Aalto University School of Science, Helsinki 00076, Finland; 5 Departament de Física Fonamental, Universitat de Barcelona, Barcelona 08028, Spain; 6 Communication and Distributed Systems group, Institute of Computer Science and Applied Mathematics, University of Bern, Bern 3012, Switzerland; 7 Palo Alto Research Center, Palo Alto, California 94304, USA; 8 Google, Mountain View, California 94043, USA; 9 Department of Physics and Texas Center for Superconductivity, University of Houston, Houston, Texas 77204, USA; 10 Max Planck Institut für Physik komplexer Systeme, Dresden 01187, Germany; 11 Department of Physics and Interdisciplinary Center for Network Science and Applications, University of Notre Dame, Notre Dame, IN 46556, USA; 12 IMT Alti Studi, Lucca 55100, Italy; 13 Department of Computer Science, Aalto University School of Science, Helsinki 00076, Finland; 14 Department of Physics, Department of Mathematics, Department of Electrical and Computer Engineering, Northeastern University, Boston, Massachusetts 02115, USA

## Abstract

Represented as graphs, real networks are intricate combinations of order and disorder. Fixing some of the structural properties of network models to their values observed in real networks, many other properties appear as statistical consequences of these fixed observables, plus randomness in other respects. Here we employ the *dk*-series, a complete set of basic characteristics of the network structure, to study the statistical dependencies between different network properties. We consider six real networks—the Internet, US airport network, human protein interactions, technosocial web of trust, English word network, and an fMRI map of the human brain—and find that many important local and global structural properties of these networks are closely reproduced by *dk*-random graphs whose degree distributions, degree correlations and clustering are as in the corresponding real network. We discuss important conceptual, methodological, and practical implications of this evaluation of network randomness, and release software to generate *dk*-random graphs.

## Abstract (web summary)

Many complex properties of real networks appear as consequences of a small set of their basic properties. Here, the authors show that *dk*-random graphs that reproduce degree distributions, degree correlations, and clustering in real networks, reproduce a variety of their other properties as well.

## Graphical abstract

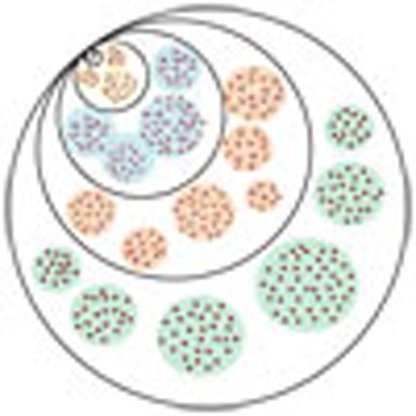

Network science studies complex systems by representing them as networks^1^. This approach has proven quite fruitful because in many cases the network representation achieves a practically useful balance between simplicity and realism: while always grand simplifications of real systems, networks often encode some crucial information about the system. Represented as a network, the system structure is fully specified by the network adjacency matrix, or the list of connections, perhaps enriched with some additional attributes. This (possibly weighted) matrix is then a starting point of research in network science.

One significant line of this research studies various (statistical) properties of adjacency matrices of real networks. The focus is often on properties that convey useful information about the global network structure that affects the dynamical processes in the system that this network represents^2^. A common belief is that a self-organizing system should evolve to a network structure that makes these dynamical processes, or network functions, efficient^3^,^4^,^5^. If this is the case, then given a real network, we may ‘reverse engineer' it by showing that its structure optimizes its function. In that respect the problem of interdependency between different network properties becomes particularly important^6^,^7^,^8^,^9^,^10^.

Indeed, suppose that the structure of some real network has property *X*—some statistically over- or under-represented subgraph, or motif^11^, for example—that we believe is related to a particular network function. Suppose also that the same network has in addition property *Y*—some specific degree distribution or clustering, for example—and that all networks that have property *Y* necessarily have property *X* as a consequence. Property *Y* thus enforces or ‘explains' property *X*, and attempts to ‘explain' *X* by itself, ignoring *Y*, are misguided. For example, if a network has high density (property *Y*), such as the interarial cortical network in the primate brain where 66% of edges that could exist do exist^12^, then it will necessarily have short path lengths and high clustering, meaning it is a small-world network (property *X*). However, unlike social networks where the small-world property is an independent feature of the network, in the brain this property is a simple consequence of high density.

The problem of interdependencies among network properties has been long understood^13^,^14^. The standard way to address it, is to generate many graphs that have property *Y* and that are random in all other respects—let us call them *Y*-random graphs—and then to check if property *X* is a typical property of these *Y*-random graphs. In other words, this procedure checks if graphs that are sampled uniformly at random from the set of all graphs that have property *Y*, also have property *X* with high probability. For example, if graphs are sampled from the set of graphs with high enough edge density, then all sampled graphs will be small worlds. If this is the case, then *X* is not an interesting property of the considered network, because the fact that the network has property *X* is a statistical consequence of that it also has property *Y*. In this case we should attempt to explain *Y* rather than *X*. In case *X* is not a typical property of *Y*-random graphs, one cannot really conclude that property *X* is interesting or important (for some network functions). The only conclusion one can make is that *Y* cannot explain *X*, which does not mean however that there is no other property *Z* from which *X* follows.

In view of this inherent and unavoidable relativism with respect to a null model, the problem of structure–function relationship requires an answer to the following question in the first place: what is the right base property or properties *Y* in the null model (*Y*-random graphs) that we should choose to study the (statistical) significance of a given property *X* in a given network^15^? For most properties *X* including motifs^11^, the choice of *Y* is often just the degree distribution. That is, one usually checks if *X* is present in random graphs with the same degree distribution as in the real network. Given that scale-free degree distributions are indeed the striking and important features of many real networks^1^, this null model choice seems natural, but there are no rigorous and successful attempts to justify it. The reason is simple: the choice cannot be rigorously justified because there is nothing special about the degree distribution—it is one of infinitely many ways to specify a null model.

Since there exists no unique preferred null model, we have to consider a series of null models satisfying certain requirements. Here we consider a particular realization of such series—the *dk*-series^16^, which provides a complete systematic basis for network structure analysis, bearing some conceptual similarities with a Fourier or Taylor series in mathematical analysis. The *dk*-series is a converging series of basic interdependent degree- and subgraph-based properties that characterize the local network structure at an increasing level of detail, and define a corresponding series of null models or random graph ensembles. These random graphs have the same distribution of differently sized subgraphs as in a given real network. Importantly, the nodes in these subgraphs are labelled by node degrees in the real network. Therefore, this random graph series is a natural generalization of random graphs with fixed average degree, degree distribution, degree correlations, clustering and so on. Using *dk*-series we analyse six real networks, and find that they are essentially random as soon as we constrain their degree distributions, correlations, and clustering to the values observed in the real network (*Y*=degrees+correlations+clustering). In other words, these basic local structural characteristics almost fully define not only local but also global organization of the considered networks. These findings have important implications on research dealing with network structure-function interplay in different disciplines where networks are used to represent complex natural or designed systems. We also find that some properties of some networks cannot be explained by just degrees, correlations, and clustering. The *dk*-series methodology thus allows one to detect which particular property in which particular network is non-trivial, cannot be reduced to basic local degree- or subgraph-based characteristics, and may thus be potentially related to some network function.

## Results

### General requirements to a systematic series of properties

The introductory remarks above instruct one to look not for a single base property *Y*, which cannot be unique or universal, but for a systematic series of base properties *Y*_0_, *Y*_1_,…. By ‘systematic' we mean the following conditions: (1) inclusiveness, that is, the properties in the series should provide strictly more detailed information about the network structure, which is equivalent to requiring that networks that have property *Y*_*d*_ (*Y*_*d*_-random graphs), *d*>0, should also have properties *Y*_*d*′_ for all *d*′=0, 1,…, *d*−1; and (2) convergence, that is, there should exist property *Y*_*D*_ in the series that fully characterizes the adjacency matrix of any given network, which is equivalent to requiring that *Y*_*D*_-random graphs is only one graph—the given network itself. If these *Y*-series satisfy the conditions above, then whatever property *X* is deemed important now or later in whatever real network, we can always standardize the problem of explanation of *X* by reformulating it as the following question: what is the minimal value of *d* in the above *Y*-series such that property *Y*_*d*_ explains *X*? By convergence, such *d* should exist; and by inclusiveness, networks that have property *Y*_*d*′_ with any *d*′=*d*, *d*+1,…, *D*, also have property *X*. Assuming that properties *Y*_*d*_ are once explained, the described procedure provides an explanation of any other property of interest *X*.

The general philosophy outlined above is applicable to undirected and directed networks, and it is shared by different approaches, including motifs^11^, graphlets^17^ and similar constructions^18^, albeit they violate the inclusiveness condition as we show below. Yet one can still define many different *Y*-series satisfying both conditions above. Some further criteria are needed to focus on a particular one. One approach is to use degree-based tailored random graphs as null models for both undirected^19^,^20^,^21^ and directed^22^,^23^ networks. The criteria that we use to select a particular *Y*-series in this study are simplicity and the importance of subgraph- and degree-based statistics in networks. Indeed, in the network representation of a system, subgraphs, their frequency and convergence are the most natural and basic building blocks of the system, among other things forming the basis of the rigorous theory of graph family limits known as graphons^24^, while the degree is the most natural and basic property of individual nodes in the network. Combining the subgraph- and degree-based characteristics leads to *dk-series*^16^.

### *dk*-series

**Figure 1:**
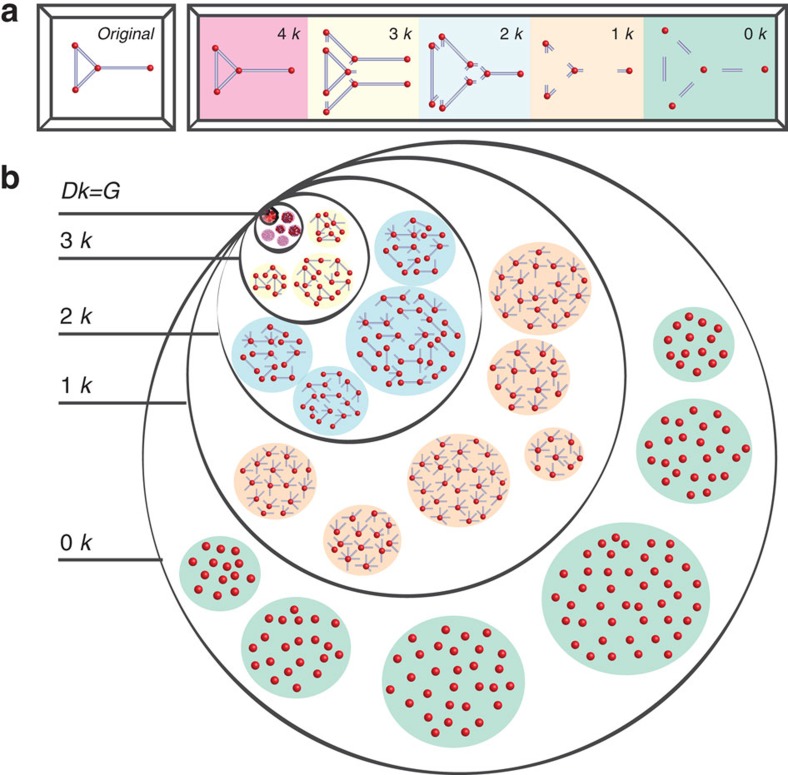
The *dk*-series illustrated. (**a**) shows the *dk*-distributions for a graph of size 4. The 4*k*-distribution is the graph itself. The 3*k*-distribution consists of its three subgraphs of size 3: one triangle connecting nodes of degrees 2, 2 and 3, and two wedges connecting nodes of degrees 2, 3 and 1. The 2*k*-distribution is the joint degree distribution in the graph. It specifies the number of links (subgraphs of size 2) connecting nodes of different degrees: one link connects nodes of degrees 2 and 2, two links connect nodes of degrees 2 and 3, and one link connects nodes of degree 3 and 1. The 1*k*-distribution is the degree distribution in the graph. It lists the number of nodes (subgraphs of size 1) of different degree: one node of degree 1, two nodes of degree 2, and one node of degree 3. The 0*k*-distribution is just the average degree in the graph, which is 2. (**b**) illustrates the inclusiveness and convergence of *dk*-series by showing the hierarchy of *dk*-graphs, which are graphs that have the same *dk*-distribution as a given graph *G* of size *D*. The black circles schematically shows the sets of *dk*-graphs. The set of 0*k*-graphs, that is, graphs that have the same average degree as *G*, is largest. Graphs in this set may have a structure drastically different from *G*'s. The set of 1*k*-graphs is a subset of 0*k*-graphs, because each graph with the same degree distribution as in *G* has also the same average degree as *G*, but not vice versa. As a consequence, typical 1*k*-graphs, that is, 1*k*-random graphs, are more similar to *G* than 0*k*-graphs. The set of 2*k*-graphs is a subset of 1*k*-graphs, also containing *G*. As *d* increases, the circles become smaller because the number of different *dk*-graphs decreases. Since all the *dk*-graph sets contain *G*, the circles ‘zoom-in' on it, and while their number decreases, *dk*-graphs become increasingly more similar to *G*. In the *d*=*D* limit, the set of *Dk*-graphs consists of only one element, *G* itself.

In *dk*-series, properties *Y*_*d*_ are *dk-distributions*. For any given network *G* of size *N*, its *dk*-distribution is defined as a collection of distributions of *G*'s subgraphs of size *d*=0, 1,…, *N* in which nodes are labelled by their degrees in *G*. That is, two isomorphic subgraphs of *G* involving nodes of different degrees—for instance, edges (*d*=2) connecting nodes of degrees 1, 2 and 2, 2—are counted separately. The 0*k*-‘distribution' is defined as the average degree of *G*. Figure 1 illustrates the *dk*-distributions of a graph of size 4.

Thus defined the *dk*-series subsumes all the basic degree-based characteristics of networks of increasing detail. The zeroth element in the series, the 0*k*-‘distribution', is the coarsest characteristic, the average degree. The next element, the 1*k*-distribution, is the standard degree distribution, which is the number of nodes—subgraphs of size 1—of degree *k* in the network. The second element, the 2*k*-distribution, is the joint degree distribution, the number of subgraphs of size 2—edges—between nodes of degrees *k*_1_ and *k*_2_. The 2*k*-distribution thus defines 2-node degree correlations and network's assortativity. For *d*=3, the two non-isomorphic subgraphs are triangles and wedges, composed of nodes of degrees *k*_1_, *k*_2_ and *k*_3_, which defines clustering, and so on. For arbitrary *d* the *dk*-distribution characterizes the ‘*d*'egree ‘*k*'orrelations in *d*-sized subgraphs, thus including, on the one hand, the correlations of degrees of nodes located at hop distances below *d*, and, on the other hand, the statistics of *d*-cliques in *G*. We will also consider *dk*-distributions with fractional *d*∈(2, 3) which in addition to specifying two-node degree correlations (*d*=2), fix some *d*=3 substatistics related to clustering.

The *dk*-series is inclusive because the (*d*+1)*k*-distribution contains the same information about the network as the *dk*-distribution, plus some additional information. In the simplest *d*=0 case for example, the degree distribution *P*(*k*) (1*k*-distribution) defines the average 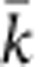 (0*k*-distribution) via 
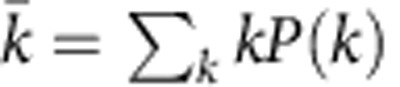
. The analogous expression for *d*=1, 2 are derived in Supplementary Note 1.

It is important to note that if we omit the degree information, and just count the number of *d*-sized subgraphs in a given network regardless their node degrees, as in motifs^11^, graphlets^17^ or similar constructions^18^, then such degree-*k*-agnostic *d*-series (versus *dk*-series) would not be inclusive (Supplementary Discussion). Therefore, preserving the node degree (‘*k*') information is necessary to make a subgraph-based (‘*d*') series inclusive. The *dk*-series is clearly convergent because at *d*=*N*, where *N* is the network size, the *Nk*-distribution fully specifies the network adjacency matrix.

A sequence of *dk*-distributions then defines a sequence of random graph ensembles (null models). The *dk-graphs* are a set of all graphs with a given *dk*-distribution, for example, with the *dk*-distribution in a given real network. The *dk*-random graphs are a maximum-entropy ensemble of these graphs^16^. This ensemble consists of all *dk*-graphs, and the probability measure is uniform (unbiased): each graph *G* in the ensemble is assigned the same probability 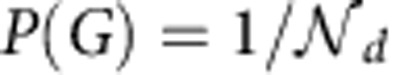, where 
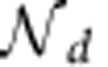
 is the number of *dk*-graphs. For *d*=0, 1, 2 these are well studied classical random graphs 
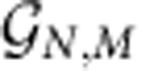
 (ref. 25), configuration model^26^,^27^,^28^ and random graphs with a given joint degree distribution^29^, respectively. Since a sequence of *dk*-distributions is increasingly more informative and thus constraining, the corresponding sequence of the sizes of *dk*-random graph ensembles is non-increasing and shrinking to 1, 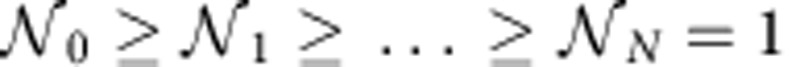, Fig. 1. At low *d*=0, 1, 2 these numbers 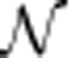 can be calculated either exactly or approximately^30^,^31^.

We emphasize that in *dk*-graphs the *dk*-distribution constraints are sharp, that is, they hold exactly—all *dk*-graphs have exactly the same *dk*-distribution. An alternative description uses soft maximum-entropy ensembles belonging to the general class of exponential random graph models^32^,^33^,^34^,^35^ in which these constraints hold only on average over the ensemble—the expected *dk*-distribution in the ensemble (not in any individual graph) is fixed to a given distribution. This ensemble consists of all possible graphs *G* of size *N*, and the probability measure *P*(*G*) is the one maximizing the ensemble entropy *S*=−∑_*G*_*P*(*G*)ln*P*(*G*) under the *dk*-distribution constraints. Using analogy with statistical mechanics, sharp and soft ensemble are often called microcanonical and canonical, respectively.

As a consequence of the convergence and inclusiveness properties of *dk*-series, any network property *X* of any given network *G* is guaranteed to be reproduced with any desired accuracy by high enough *d*. At *d*=*N* all possible properties are reproduced exactly, but the *Nk*-graph ensemble trivially consists of only one graph, *G*self, and has zero entropy. In the sense that the entropy of *dk*-ensembles 
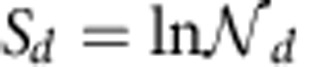
 is a non-increasing function of *d*, the smaller the *d*, the more random the *dk*-random graphs, which also agrees with the intuition that *dk*-random graphs are ‘the less random and the more structured', the higher the *d*. Therefore, the general problem of explaining a given property *X* reduces to the general problem of how random a graph ensemble must be so that *X* is statistically significant. In the *dk*-series context, this question becomes: how much local degree information, that is, information about concentrations of degree-labelled subgraphs of what minimal size *d*, is needed to reproduce a possibly global property *X* with a desired accuracy?

Below we answer this question for a set of popular and commonly used structural properties of some paradigmatic real networks. But to answer this question for any property in any network, we have to be able to sample graphs uniformly at random from the sets of *dk*-graphs—the problem that we discuss next.

### *dk*-random graph sampling

Soft *dk*-ensembles tend to be more amenable for analytic treatment, compared with sharp ensembles, but even in soft ensembles the exact analytic expressions for expected values are known only for simplest network properties in simplest ensembles^36^,^37^. Therefore, we retreat to numeric experiments here. Given a real network *G*, there exist two ways to sample *dk*-random graphs in such experiments: *dk*-randomize *G* generalizing the randomization algorithms in refs 38, 39, or construct random graphs with *G*'s *dk*-sequence from scratch^16^,^40^, also called direct construction^41^,^42^,^43^,^44^.

**Figure 2:**
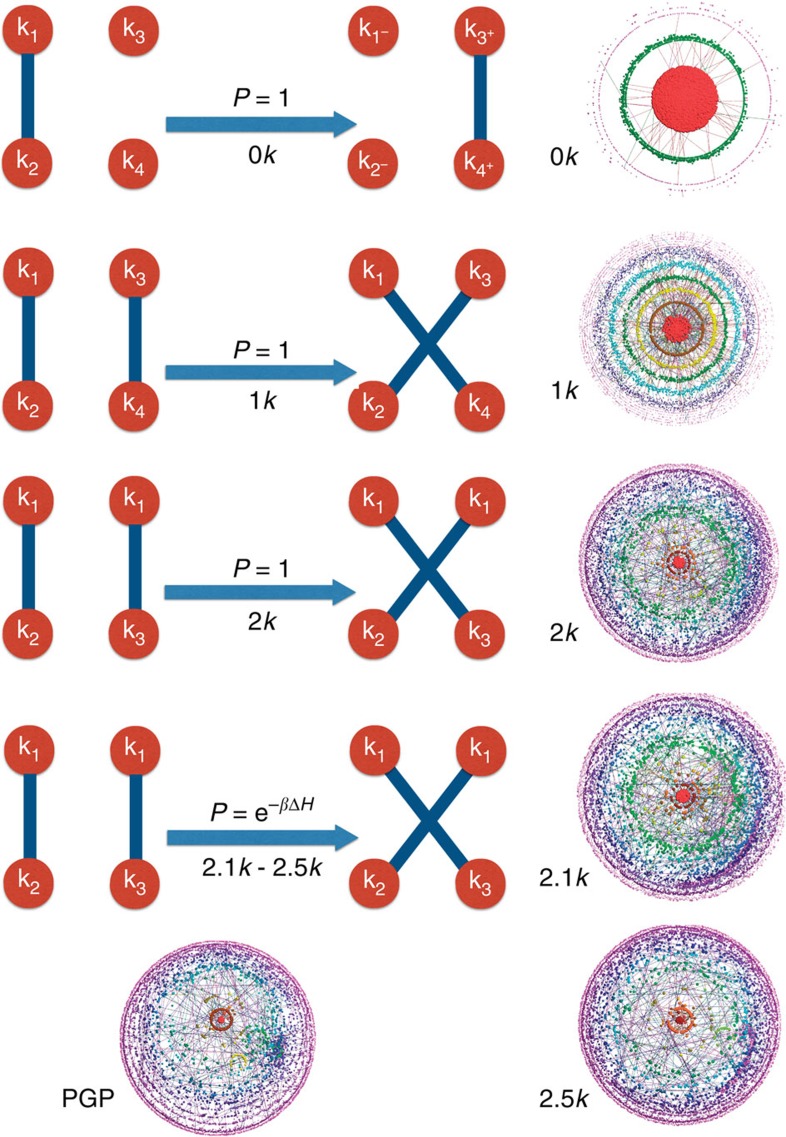
The *dk*-sampling and convergence of *dk*-series illustrated. The left column shows the elementary swaps of *dk*-randomizing (for *d*=0, 1, 2) and *dk*-targeting (for *d*=2.1, 2.5) rewiring. The nodes are labelled by their degrees, and the arrows are labelled by the rewiring acceptance probability. In *dk*-randomizing rewiring, random (pairs of) edges are rewired preserving the graph's *dk*-distribution (and consequently its *d*′*K*-distributions for all *d*′<*d*). In 2.1*k*- and 2.5*k*-targeting rewiring, the moves preserve the 2*k*-distribution, but each move is accepted with probability *p* designed to drive the graph closer to a target value of average clustering 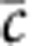 (2.1*k*) or degree-dependent clustering (*k*) (2.5*k*): *p*=min(1, *e*^−*β*Δ*H*^), where β the inverse temperature of this simulated annealing process, Δ*H*=*H*_*a*_−*H*_*b*_, and *H*_*a*,*b*_ are the distances, after and before the move, between the current and target values of clustering: 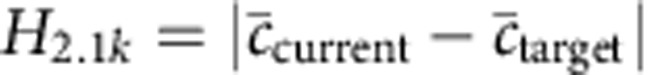 and 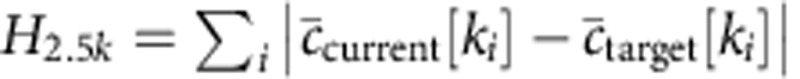. The right column shows LaNet-vi (ref. 65) visualizations of the results of these *dk*-rewiring processes (Supplementary Methods), applied to the PGP network, visualized at the bottom of the left column. The node sizes are proportional to the logarithm of their degrees, while the colour reflects node coreness^65^. As *d* grows, the shown *dk*-random graphs quickly become more similar to the real PGP network.

The first option, *dk*-randomization, is easier. It accounts for swapping random (pairs of) edges, starting from *G*, such that the *dk*-distribution is preserved at each swap, Fig. 2. There are many concerns with this prescription^45^, two of which are particularly important. The first concern is if this process ‘ergodic', meaning that if any two *dk*-graphs are connected by a chain of *dk*-swaps. For *d*=1 the two-edge swap is ergodic^38^,^39^, while for *d*=2 it is not ergodic. However, the so-called restricted two-edge swap, when at least one node attached to each edge has the same degree, Fig. 2, was proven to be ergodic^46^. It is now commonly believed that there is no edge-swapping operation, of this or other type, that is ergodic for the 3*k*-distribution, although a definite proof is lacking at the moment. If there exists no ergodic 3*k*-swapping, then we cannot really rely on it in sampling *dk*-random graphs because our real network *G* can be trapped on a small island of atypical *dk*-graphs, which is not connected by any *dk*-swap chain to the main land of many typical *dk*-graphs. Yet we note that in an unpublished work^47^ we showed that five out of six considered real networks were virtually indistinguishable from their 3*k*-randomizations across all the considered network properties, although one network (power grid) was very different from its 3*k*-random counterparts.

The second concern with *dk*-randomization is about how close to uniform sampling the *dk*-swap Markov chain is after its mixing time is reached—its mixing time is yet another concern that we do not discuss here, but according to many numerical experiments and some analytic estimates, it is *O*(*M*)^16^,^29^,^38^,^39^,^40^,^46^. Even for *d*=1 the swap chain does not sample 1*k*-graphs uniformly at random, yet if the edge-swap process is done correctly, then the sampling is uniform^20^,^21^.

A simple algorithm for the second *dk*-sampling option, constructing *dk*-graphs from scratch, is widely known for *d*=1: given *G*'s degree sequence {*k*_*i*_}, build a 1*k*-random graph by attaching *k*_*i*_ half-edges (‘stubs') to node *i*, and then connect random pairs of stubs to form edges^27^. If during this process a self-loop (both stubs are connected to the same node) or double-edge (two edges between the same pair of nodes) is formed, one has to restart the process from scratch since otherwise the graph sampling is not uniform^48^. If the degree sequence is power-law distributed with exponent close to −2 as in many real networks, then the probability that the process must be restarted approaches 1 for large graphs^49^, so that this construction process never succeeds. An alternative greedy algorithm is described in ref. 42, which always quickly succeeds and gives an efficient way of testing whether a given sequence of integers is graphical, that is, whether it can be realized as a degree sequence of a graph. The base sampling procedure does not sample graphs uniformly, but then an importance sampling procedure is used to account for the bias, which results in uniform sampling. Yet again, if the degree distribution is a power law, then one can show that even without importance sampling, the base sampling procedure is uniform, since the distribution of sampling weights that one can compute for this greedy algorithm approaches a delta function. Extensions of the naive 1*k*-construction above to 2*k* are less known, but they exist^16^,^29^,^44^,^50^. Most of these 2*k*-constructions do not sample 2*k*-graphs exactly uniformly either^46^, but importance sampling^20^,^44^ can correct for the sampling biases.

Unfortunately, to the best of our knowledge, there currently exists no 3*k*-construction algorithm that can be successfully used in practice to generate large 3*k*-graphs with 3*k*-distributions of real networks. The 3*k*-distribution is quite constraining and non-local, so that the *dk*-construction methods described above for *d*=1, 2 cannot be readily extended to *d*=3 (ref. 16). There is yet another option—3*k*-targeting rewiring, Fig. 2. It is 2*k*-preserving rewiring in which each 2*k*-swap is accepted not with probability 1, but with probability equal to min(1, exp(−*β*Δ*H*)), where β is the inverse temperature of this simulated-annealing-like process, and Δ*H* is the change in the *L*^1^ distance between the 3*k*-distribution in the current graph and the target 3*k*-distribution before and after the swap. This probability favours and, respectively, suppresses 2*k*-swaps that move the graph closer or farther from the target 3*k*-distribution. Unfortunately, we report that in agreement with^40^ this 2*k*-preserving 3*k*-targeting process never converged for any considered real network—regardless of how long we let the rewiring code run, after the initial rapid decrease, the 3*k*-distance, while continuing to slowly decrease, remained substantially large. The reason why this process never converges is that the 3*k*-distribution is extremely constraining, so that the number of 3*k*-graphs 
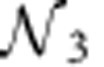
 is infinitesimally small compared with the number of 2*k*-graphs 
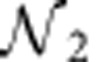
, 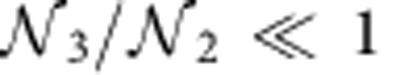 (refs 16, 30). Therefore, it is extremely difficult for the 3*k*-targeting Markov chain to find a rare path to the target 3*k*-distribution, and the process gets hopelessly trapped in abundant local minima in distance *H*.

Therefore, on one hand, even though 3*k*-randomized versions of many real networks are indistinguishable from the original networks across many metrics^47^, we cannot use this fact to claim that at *d*=3 these metrics are not statistically significant in those networks, because the 3*k*-randomization Markov chain may be non-ergodic. On the other hand, we cannot generate the corresponding 3*k*-random graphs from scratch in a feasible amount of compute time. The 3*k*-random graph ensemble is not analytically tractable either. Given that *d*=2 is not enough to guarantee the statistical insignificance of some important properties of some real networks, see ref. 47 and below, we, as in ref. 40, retreat to numeric investigations of 2*k*-random graphs in which in addition to the 2*k*-distribution, some substatistics of the 3*k*-distribution is fixed. Since strong clustering is a ubiquitous feature of many real networks^1^, one of the most interesting such substatistics is clustering.

Specifically we study 2.1*k*-random graphs, defined as 2*k*-random graphs with a given value of average clustering 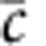, and 2.5*k*-random graphs, defined as 2*k*-random graphs with given values of average clustering 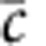
(*k*) of nodes of degree *k* (ref. 40). The 3*k*-distribution fully defines both 2.1*k*- and 2.5*k*-statistics, while 2.5*k* defines 2.1*k*. Therefore, 2*k*-graphs are a superset of 2.1*k*-graphs, which are a superset of 2.5*k*-graphs, which in turn contain all the 3*k*-graphs, 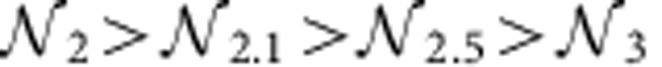. Therefore if a particular property is not statistically significant in 2.5*k*-random graphs, for example, then it is not statistically significant in 3*k*-random graphs either, while the converse is not generally true.

We thus generate 20 *dk*-random graphs with *d*=0, 1, 2, 2.1, 2.5 for each considered real network. For *d*=0,1,2 we use the standard *dk*-randomizing swapping, Fig. 2. We do not use its modifications to guarantee exactly uniform sampling^20^,^21^, because: (1) even without these modifications the swapping is close to uniform in power-law graphs, (2) these modifications are non-trivial to efficiently implement, and (3) we could not extend these modifications to the 2.1*k* and 2.5*k* cases. As a consequence, our sampling is not exactly uniform, but we believe it is close to uniform for the reasons discussed above. To generate *dk*-random graphs with *d*=2.1, 2.5, we start with a 2*k*-random graph, and apply to it the standard 2*k*-preserving 2.*xk*-targeting (*x*=1, 5) rewiring process, Fig. 2. The algorithms that do that, as described in ref. 40, did not converge on some networks, so that we modified the algorithm in ref. 10 to ensure the convergence in all cases. The details of these modifications are in Supplementary Methods (the parameters used are listed in Supplementary Table 4), along with the details of the software package implementing these algorithms that we release to public^51^.

### Real versus *dk*-random networks

**Figure 3:**
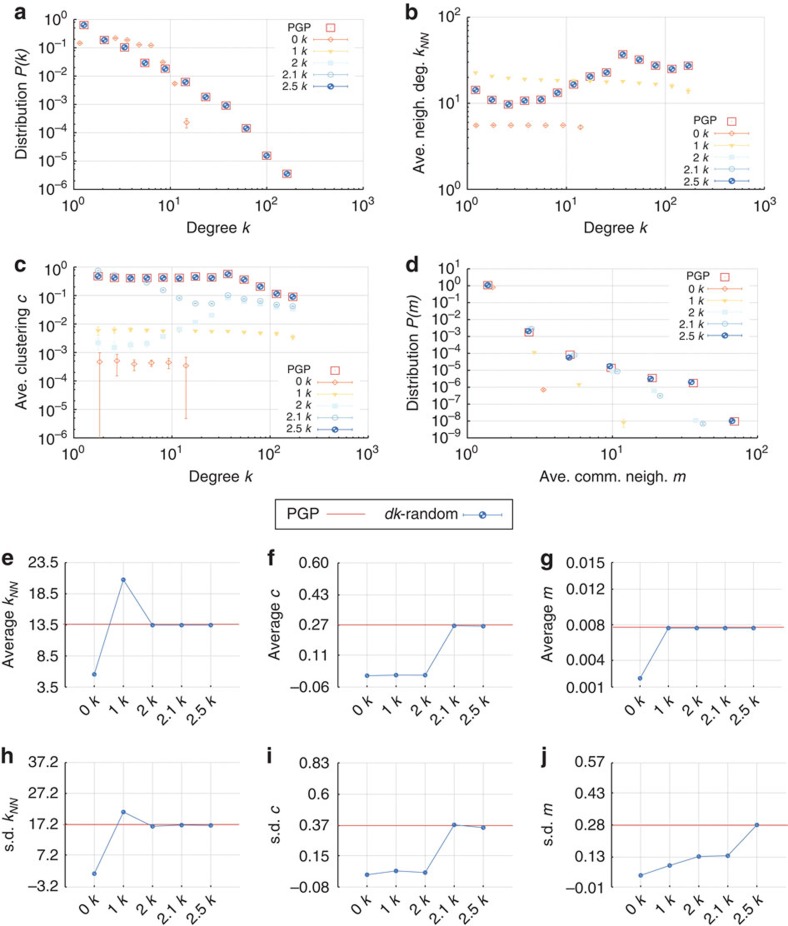
Microscopic properties of the PGP network and its *dk*-random graphs. (**a**) The degree distribution *P*(*k*), (**b**) average degree 
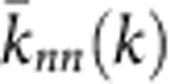
 of nearest neighbours of nodes of degree *k*, (**c**) average clustering 
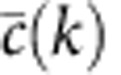
 of nodes of degree *k*, (**d**) the distribution *P*(*m*) of the number *m* of common neighbours between all connected pairs of nodes, and (**e**–**g**) the means and (**h**–**j**) s.d. of the corresponding distributions. The error bars indicate the s.d. of the metrics across different graph realizations.

**Figure 4:**
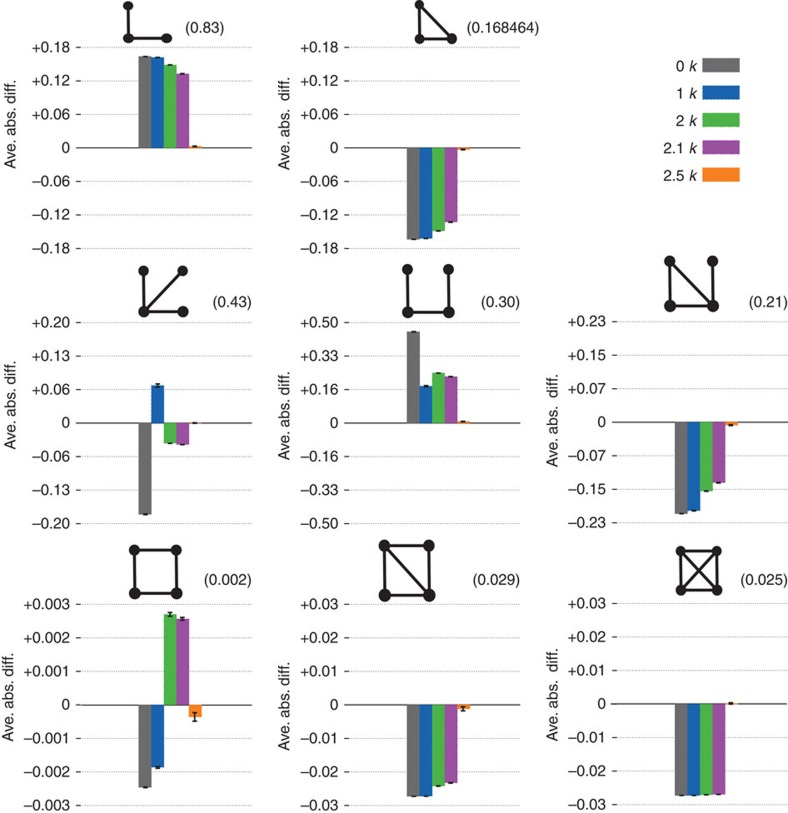
The densities of subgraphs of size 3 and 4 in the PGP network and its *dk*-random graphs. The two different graphs of size 3 and six different graphs of size 4 are shown on each panel. The numbers on top of panels are the concentrations of the corresponding subgraph in the PGP network, while the histogram heights indicate the average absolute difference between the subgraph concentration in the *dk*-random graphs and its concentration in the PGP network. The subgraph concentration is the number of given subgraphs divided by the total number of subgraphs of the same size. The error bars are the s.d. across different graph realizations.

**Figure 5:**
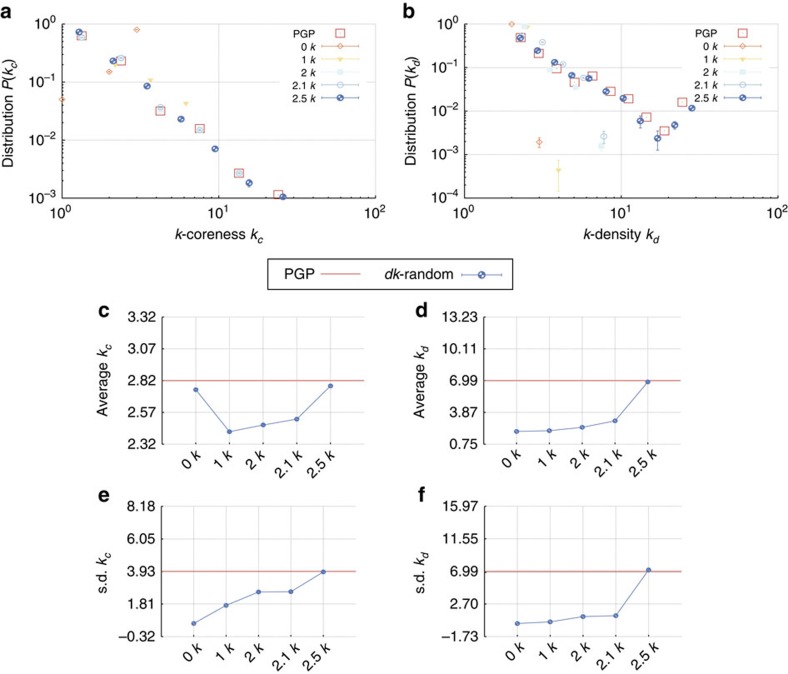
Mesoscopic properties, the *k*-coreness and *k*-density distributions, in the PGP network and its *dk*-random graphs. The figure shows the distributions *P*(*k*_*c*,*d*_) of (**a**) node *k*-coreness *k*_*c*_ and (**b**) edge *k*-density *k*_*d*_, and their (**c**,**d**) means and (**e**,**f**) s.d. The *k*_*c*_-core of a graph is its maximal subgraph in which all nodes have degree at least *k*_*c*_. The *k*_*d*_-core of a graph is its maximal subgraph in which all edges have multiplicity at least *k*_*d*_−2; the multiplicity of an edge is the number of common neighbours between the nodes that this edge connects, or equivalently the number of triangles that this edge belongs to. A node has *k*-coreness *k*_*c*_ if it belongs to the *k*_*c*_-core but not to the *k*_*c*_+1-core. An edge has *k*-density *k*_*d*_ if it belongs to the *k*_*d*_-core but not to the *k*_*d*_+1-core. The error bars indicate the s.d. of the metrics across different graph realizations.

**Figure 6:**
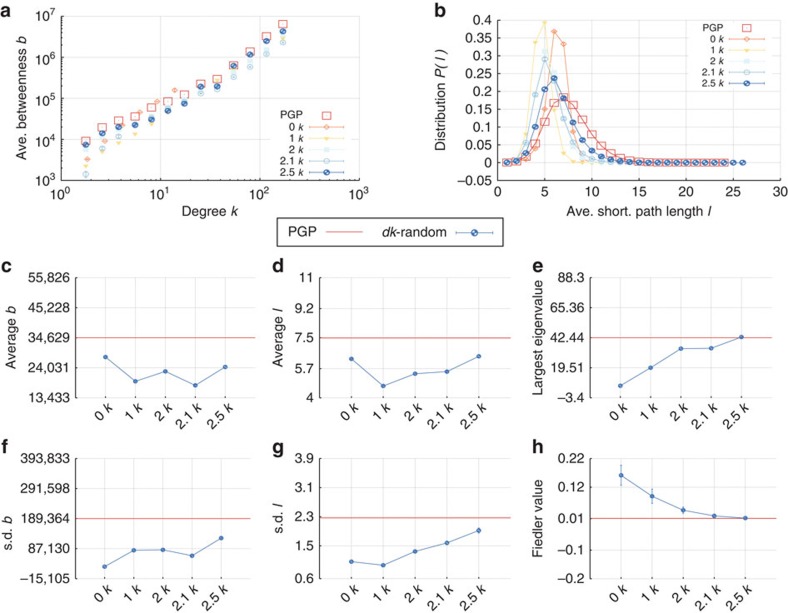
Macroscopic properties of the PGP network and its *dk*-random graphs. (**a**) The average betweenness 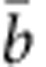
(*k*) of nodes of degree *k*, (**b**) the distribution *P*(*l*) of hop lengths *l* of the shortest paths between all pairs of nodes, the (**c**,**d**) means and (**f**,**g**) s.d. of the corresponding distributions, (**e**) the largest eigenvalues of the adjacency matrix *A*, and (**h**) the Fiedler value, which is the spectral gap (the second largest eigenvalue) of the graph's Laplacian matrix *L*=*D*−*A*, where *D* is the degree matrix, *D*_*ij*_=*δ*_*ij*_*k*_*i*_, *δ*_*ij*_ the Kronecker delta, and *k*_*i*_ the degree of node *i*. The error bars indicate the s.d. of the metrics across different graph realizations.

We performed an extensive set of numeric experiments with six real networks—the US air transportation network, an fMRI map of the human brain, the Internet at the level of autonomous systems, a technosocial web of trust among users of the distributed Pretty Good Privacy (PGP) cryptosystem, a human protein interaction map, and an English word adjacency network (Supplementary Note 2 and Supplementary Table 3 present the analysed networks). For each network we compute its average degree, degree distribution, degree correlations, average clustering, averaging clustering of nodes of degree *k* and based on these *dk*-statistics generate a number of *dk*-random graphs as described above for each *d*=0, 1, 2, 2.1, 2.5. Then for each sample we compute a variety of network properties, and report their means and deviations for each combination of the real network, *d*, and the property. Figures 3, 4, 5, 6 present the results for the PGP network; Supplementary Note 3, Supplementary Figs 1–10, and Supplementary Tables 1–2 provide the complete set of results for all the considered real networks. The reason why we choose the PGP network as our main example is that this network appears to be ‘least random' among the considered real networks, in the sense that the PGP network requires higher values of *d* to reproduce its considered properties. The only exception is the brain network. Some of its properties are not reproduced even by *d*=2.5.

Figure 2 visualizes the PGP network and its *dk*-randomizations. The figure illustrates the convergence of *dk*-series applied to this network. While the 0*k*-random graph has very little in common with the real network, the 1*k*-random one is somewhat more similar, even more so for 2*k*, and there is very little visual difference between the real PGP network and its 2.5*k*-random counterpart. This figure is only an illustration though, and to have a better understanding of how similar the network is to its randomization, we compare their properties.

We split the properties that we compare into the following categories. The microscopic properties are local properties of individual nodes and subgraphs of small size. These properties can be further subdivided into those that are defined by the *dk*-distributions—the degree distribution, average neighbour degree, clustering, Fig. 3—and those that are not fixed by the *dk*-distributions—the concentrations of subgraphs of size 3 and 4, Fig. 4. The mesoscopic properties—*k*-coreness and *k*-density (the latter is also known as *m*-coreness or edge multiplicity, Supplementary Note 1), Fig. 5—depend both on local and global aspects of network organization. Finally, the macroscopic properties are truly global ones—betweenness, the distribution of hop lengths of shortest paths, and spectral properties, Fig. 6. In Supplementary Note 3 we also report some extremal properties, such as the graph diameter (the length of the longest shortest path), and Kolmogorov–Smirnov distances between the distributions of all the considered properties in real networks and their corresponding *dk*-random graphs. The detailed definitions of all the properties that we consider can be found in Supplementary Note 1.

In most cases—henceforth by ‘case' we mean a combination of a real network and one of its considered property—we observe a nice convergence of properties as *d* increases. In many cases there is no statistically significant difference between the property in the real network and in its 2.5*k*-random graphs. In that sense these graphs, that is, random graphs whose degree distribution, degree correlations, and degree-dependent clustering 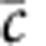
(*k*) are as in the original network, capture many other important properties of the real network.

Some properties always converge. This is certainly true for the microscopic properties in Fig. 3, simply confirming that our *dk*-sampling algorithm operates correctly. But many properties that are not fixed by the *dk*-distributions converge as well. Neither the concentration of subgraphs of size 3 nor the distribution of the number of neighbours common to a pair of nodes are fully fixed by *dk*-distributions with any *d*<3 by definition, yet 2.5*k*-random graphs reproduce them well in all the considered networks. Most subgraphs of size 4 are also captured at *d*=2.5 in most networks, even though *d*=3 would not be enough to exactly reproduce the statistics of these subgraphs. We note that the improvement in subgraph concentrations at *d*=2.5 compared with *d*=2.1 is particularly striking, Fig. 4. The mesoscopic and especially macroscopic properties converge more slowly as expected. Nevertheless, quite surprisingly, both mesoscopic properties (*k*-coreness and *k*-density) and some macroscopic properties converge nicely in most cases. The *k*-coreness, *k*-density, and the spectral properties, for instance, converge at *d*=2.5 in all the considered cases other than Internet's Fiedler value. In some cases a property, even global one, converges for *d* <2.5. Betweenness, for example, a global property, converges at *d*=1 for the Internet and English word network.

Finally, there are ‘outlier' networks and properties of poor or no *dk*-convergence. Many properties of the brain network, for example, exhibit slow or no convergence. We have also experimented with community structure inferred by different algorithms, and in most cases the convergence is either slow or non-existent as one could expect.

## Discussion

In general, we should not expect non-local properties of networks to be exactly or even closely reproduced by random graphs with local constraints. The considered brain network is a good example of that this expectation is quite reasonable. The human brain consists of two relatively weakly connected parts, and no *dk*-randomization with low *d* is expected to reproduce this peculiar global feature, which likely has an impact on other global properties. And indeed we observe in Supplementary Note 3 that its two global properties, the shortest path distance and betweenness distributions, differ drastically between the brain and its *dk*-randomizations.

Another good example is community structure, which is not robust with respect to *dk*-randomizations in all the considered networks. In other words, *dk*-randomizations destroy the original peculiar cluster organization in real networks, which is not surprising, as clusters have too many complex non-local features such as variable densities of internal links, boundaries and so on, which *dk*-randomizations, even with high *d*, are expected to affect considerably.

Surprisingly, what happens for the brain and community structure does not appear representative for many other considered combinations of real networks and their properties. As a possible explanation, one can think of constraint-based modelling as a satisfiability (SAT) problem: find the elements of the adjacency matrix (1/0, True/False) such that all the given constraints in terms of the functions of the marginals (degrees) of this matrix are obeyed. We then expect that the 3*k*-constraints already correspond to an NP-hard SAT problem, such as 3-SAT, with hardness coming from the global nature of the constraints in the problem. However, many real-world networks evolve based mostly on local dynamical rules and thus we would expect them to contain correlations with *d*<3, that is, below the NP-hard barrier. The primate brain, however, has likely evolved through global constraints, as indicated by the dense connectivity across all functional areas and the existence of a strong core-periphery structure in which the core heavily concentrates on areas within the associative cortex, with connections to and from all the primary input and subcortical areas^12^.

However, in most cases, the considered networks are *dk*-random with *d*≤2.5, that is, *d*≤2.5 is enough to reproduce not only basic microscopic (local) properties but also mesoscopic and even macroscopic (global) network properties^6^,^7^,^8^,^9^,^10^. This finding means that these more sophisticated properties are effectively random in the considered networks, or more precisely, that the observed values of these properties are effective consequences of particular degree distributions and, optionally, degree correlations and clustering that the networks have. This further implies that attempts to find explanations for these complex but effectively random properties should probably be abandoned, and redirected to explanations of why and how degree distributions, correlations and clustering emerge in real networks, for which there already exists a multitude of approaches^52^,^53^,^54^,^55^,^56^,^57^. On the other hand, the features that we found non-random do require separate explanations, or perhaps a different system of null models.

We reiterate that the *dk*-randomization system makes it clear that there is no *a priori* preferred null model for network randomization. To tell how statistically significant a particular feature is, it is necessary to compare this feature in the real network against the same feature in an ensemble of random graphs, a null model. But one is free to choose any random graph model. In particular, any *d* defines a random graph ensemble, and we find that many properties, most notably the frequencies of small subgraphs that define motifs^11^, strongly depend on *d* for many considered networks. Therefore, choosing any specific value of *d*, or more generally, any specific null model to study the statistical significance of a particular structural network feature, requires some non-trivial justification before this feature can be claimed important for any network function.

Yet another implication of our results is that if one looks for network topology generators that would veraciously reproduce certain properties of a given real network—a task that often comes up in as diverse disciplines as biology^58^ and computer science^59^—one should first check how *dk*-random these properties are. If they are *dk*-random with low *d*, then one may not need any sophisticated mission-specific topology generators. The *dk*-random graph-generation algorithms discussed here can be used for that purpose in this case. We note that there exists an extension of a subset of these algorithm for networks with arbitrary annotations of links and nodes^60^—directed or coloured (multilayer) networks, for instance.

The main caveat of our approach is that we have no proof that our *dk*-random graph generation algorithms for *d*=2.1 and *d*=2.5 sample graphs uniformly at random from the ensemble. The random-graph ensembles and edge-rewiring processes employed here are known to suffer from problems such as degeneracy and hysteresis^35^,^61^,^62^. Ideally, we would wish to calculate analytically the exact expected value of a given property in an ensemble. This is currently possible only for very simple properties in soft ensembles with *d*=0, 1, 2 (refs 36, 37). Some mathematically rigorous results are available for *d*=0, 1 and for some exponential random-graph models^28^,^34^. Many of these results rely on graphons^24^ that are applicable to dense graphs only, while virtually all real networks are sparse^49^. Some rigorous approaches to sparse networks are beginning to emerge^63^,^64^, but the rigorous treatment of global properties, which tend to be highly non-trivial functions of adjacency matrices, in random graph ensembles with *d*>2 constraints, appear to be well beyond the reach in the near future. Yet if we ever want to fully understand the relationship between the structure, function and dynamics of real networks, this future research direction appears to be of a paramount importance.

## Additional information

**How to cite this article:** Orsini, C. *et al*. Quantifying randomness in real networks. *Nat. Commun.* 6:8627 doi: 10.1038/ncomms9627 (2015).

## Supplementary Material

Supplementary Information Supplementary Figures 1-10, Supplementary Tables 1-5, Supplementary Notes 1-3, Supplementary Discussion, Supplementary Methods and Supplementary References
