# Supplementary material for: Quantifying randomness in real networks
Source: Nat Commun. 2015 Oct 20;6:8627. doi: 10.1038/ncomms9627 (PMC4667701; doi:10.1038/ncomms9627)
Supplement: Supplementary Information — Supplementary Figures 1-10, Supplementary Tables 1-5, Supplementary Notes 1-3, Supplementary Discussion, Supplementary Methods and Supplementary References [file ncomms9627-s1.pdf]

Supplementary Information

Supplementary Figures

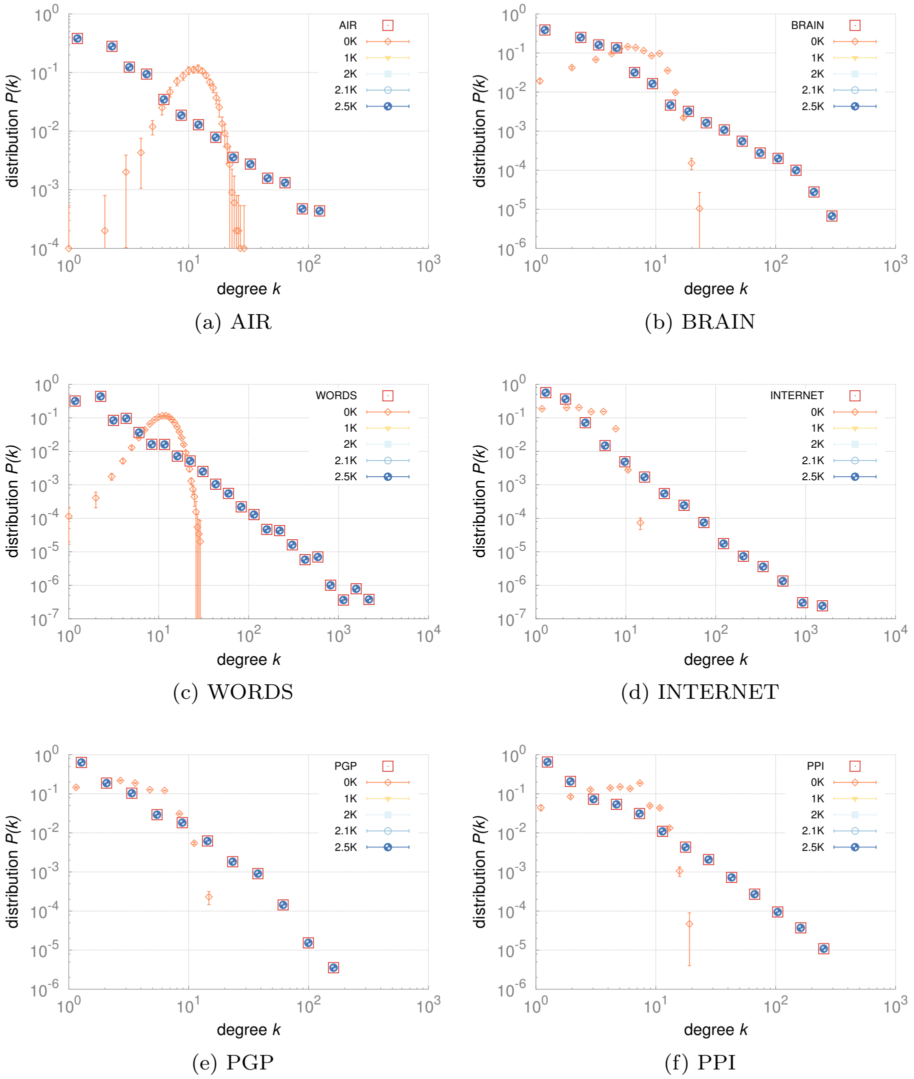

(a) AIR (b) BRAIN

(c) WORDS (d) INTERNET

(e) PGP (f) PPI

Supplementary Figure 1: **Degree distributions in real networks and their** dk**-randomizations.**

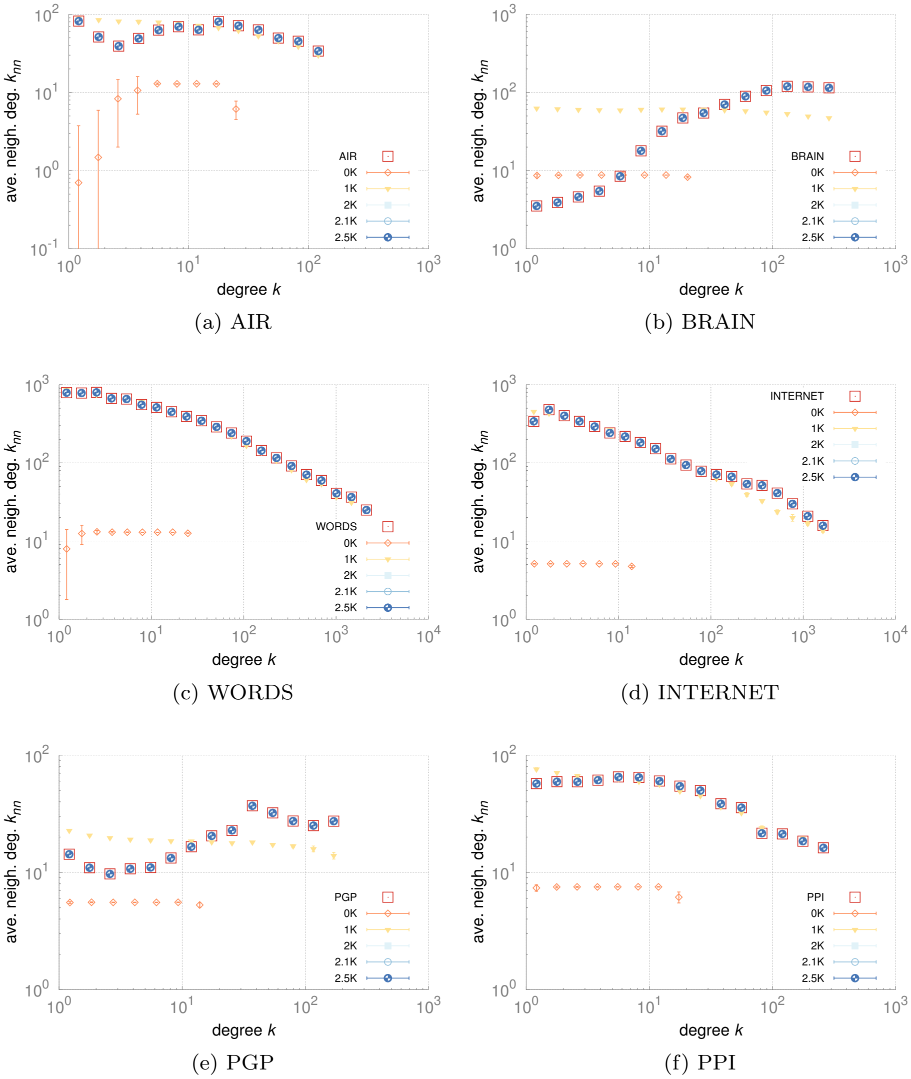

(a) AIR (b) BRAIN

(c) WORDS (d) INTERNET

(e) PGP (f) PPI

Supplementary Figure 2: **Average nearest neighbor degrees (ANNDs) of nodes of a given degree in real networks and their dk-randomizations.**

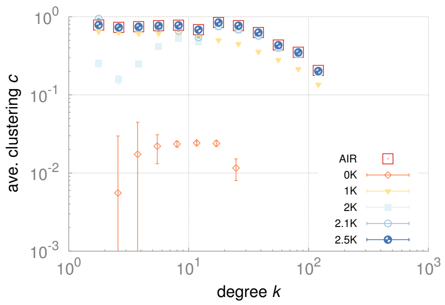

(a) AIR

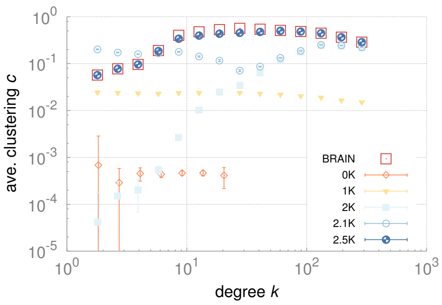

(b) BRAIN

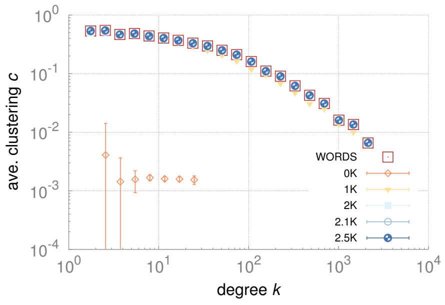

(c) WORDS

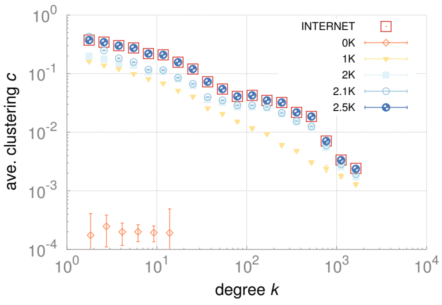

(d) INTERNET

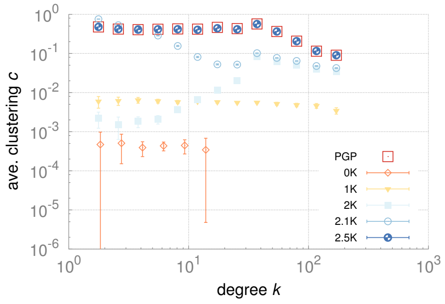

(e) PGP

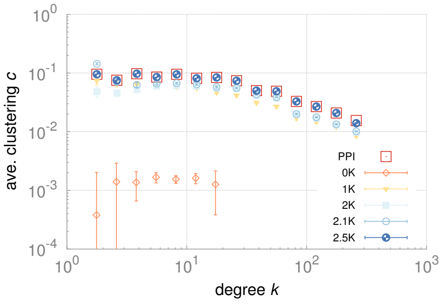

(f) PPI

Supplementary Figure 3: **Average clustering of nodes of a given degree in real networks and their dk-randomizations.**

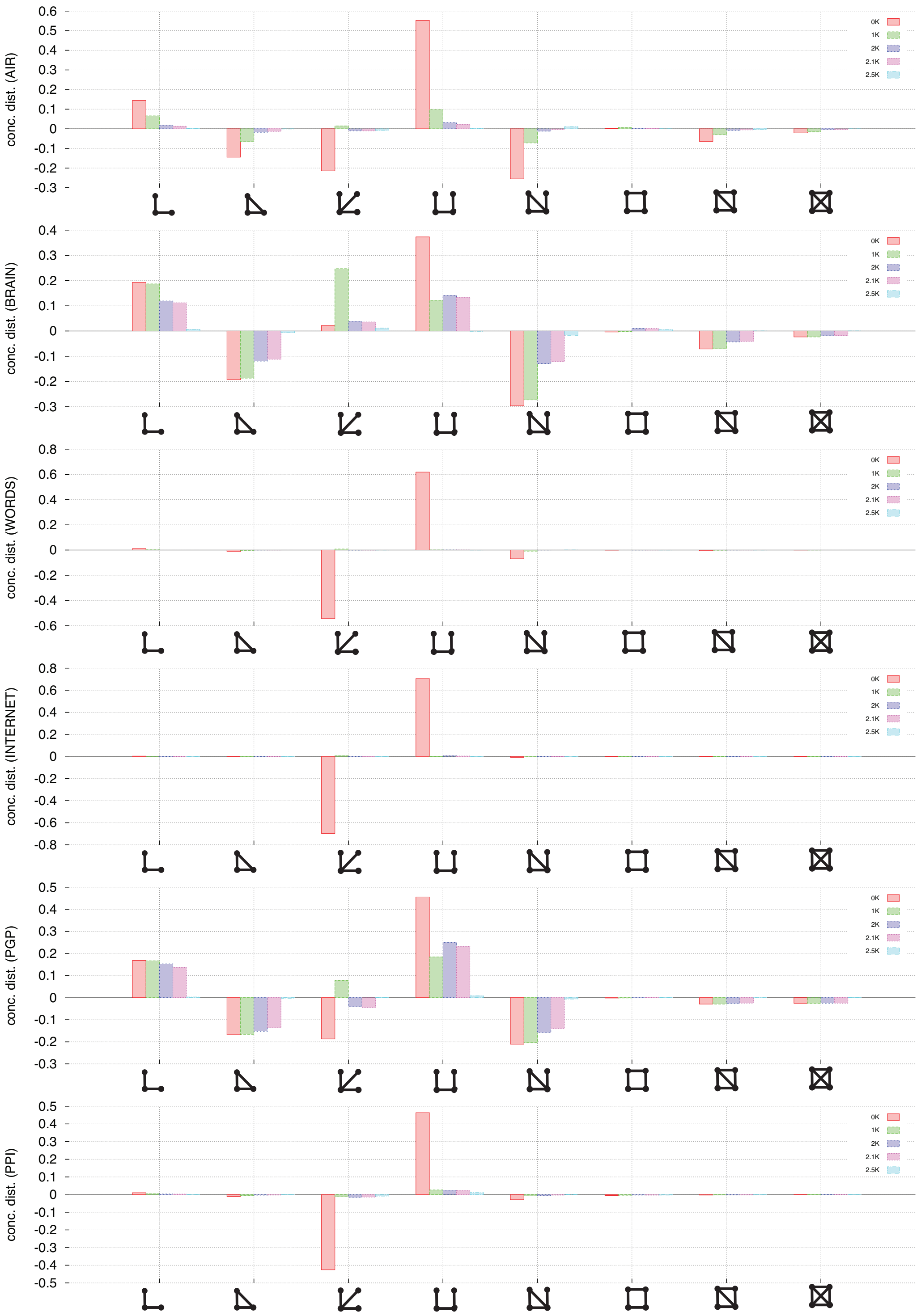

Supplementary Figure 4: **Subgraph concentration differences betweesn dk-randomizations and real networks.**

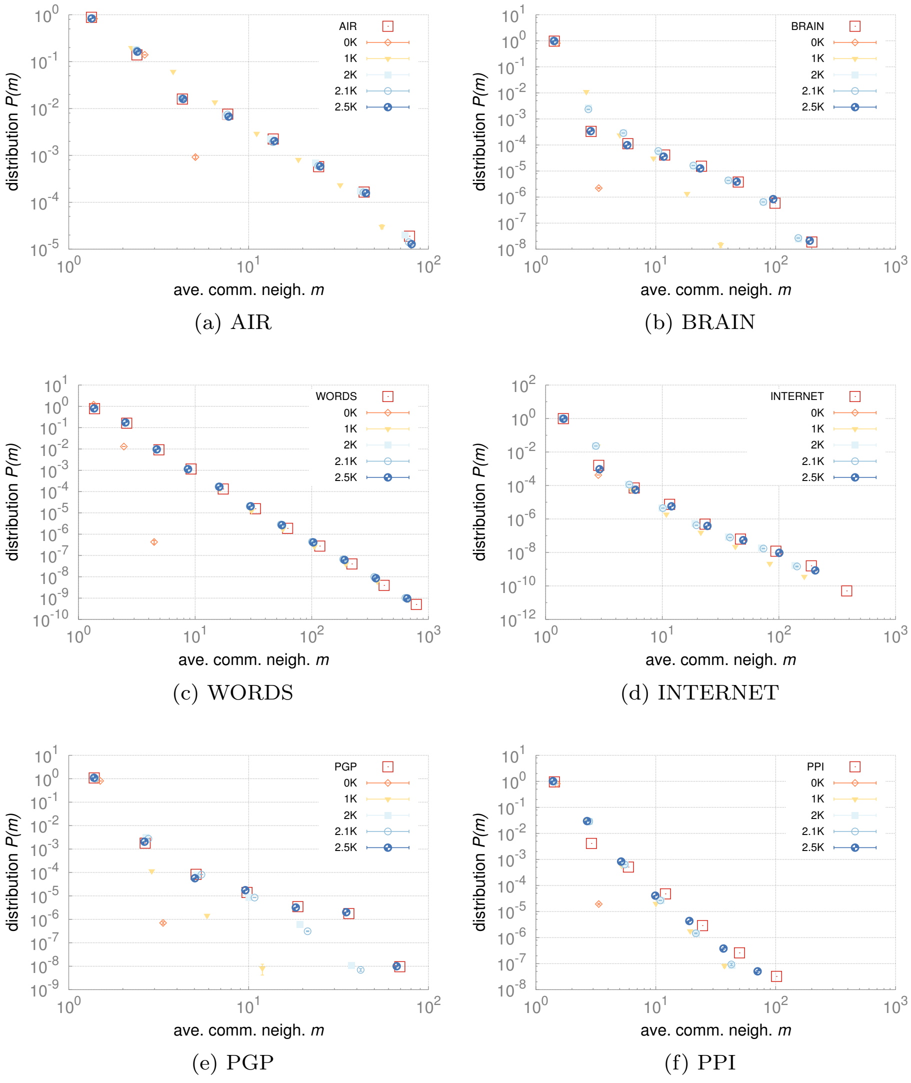

(a) AIR (b) BRAIN

(c) WORDS (d) INTERNET

(e) PGP (f) PPI

Supplementary Figure 5: **Common neighbor distributions in real networks and their dk-randomizations.**

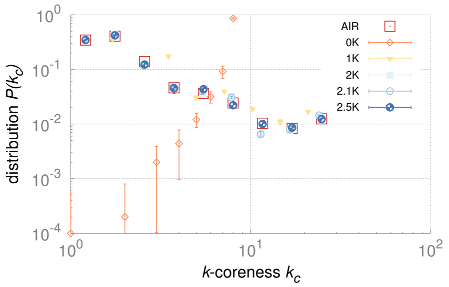

(a) AIR

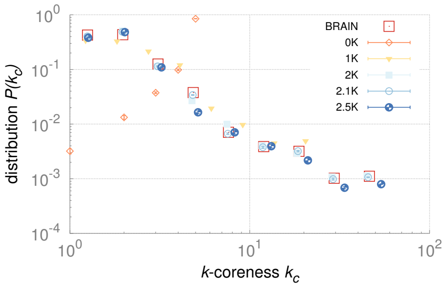

(b) BRAIN

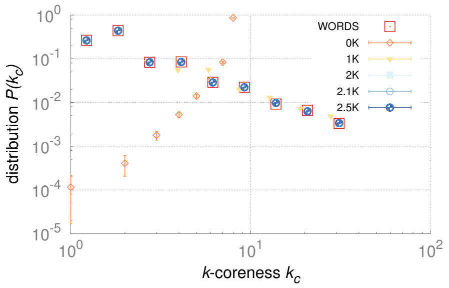

(c) WORDS

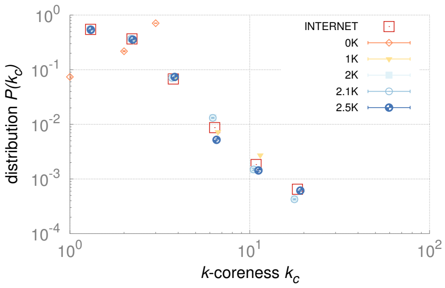

(d) INTERNET

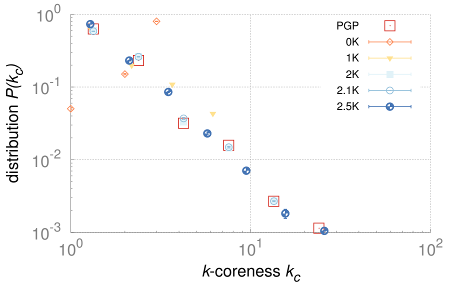

(e) PGP

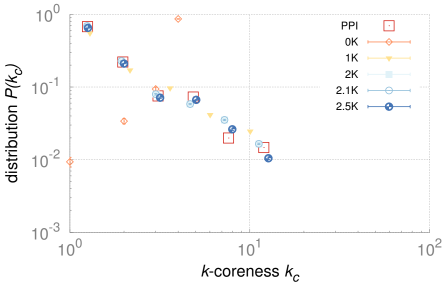

(f) PPI

Supplementary Figure 6: **k-coreness distributions in real networks and their dk-randomizations.**

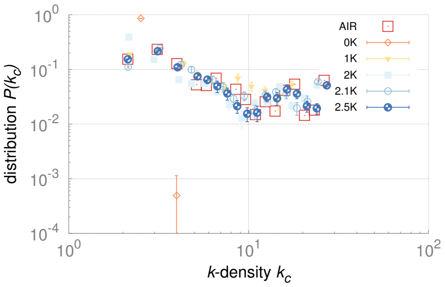

(a) AIR

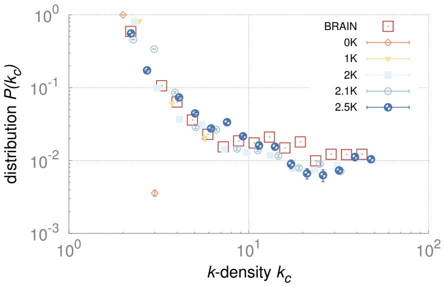

(b) BRAIN

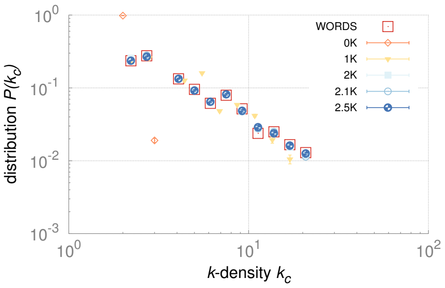

(c) WORDS

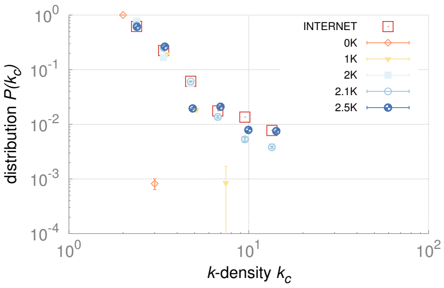

(d) INTERNET

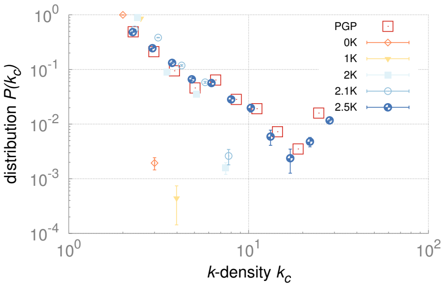

(e) PGP

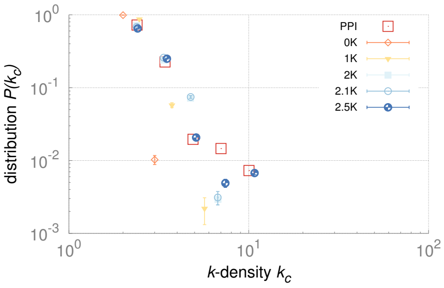

(f) PPI

Supplementary Figure 7: **k-denseness distributions in real networks and their dk-randomizations.**

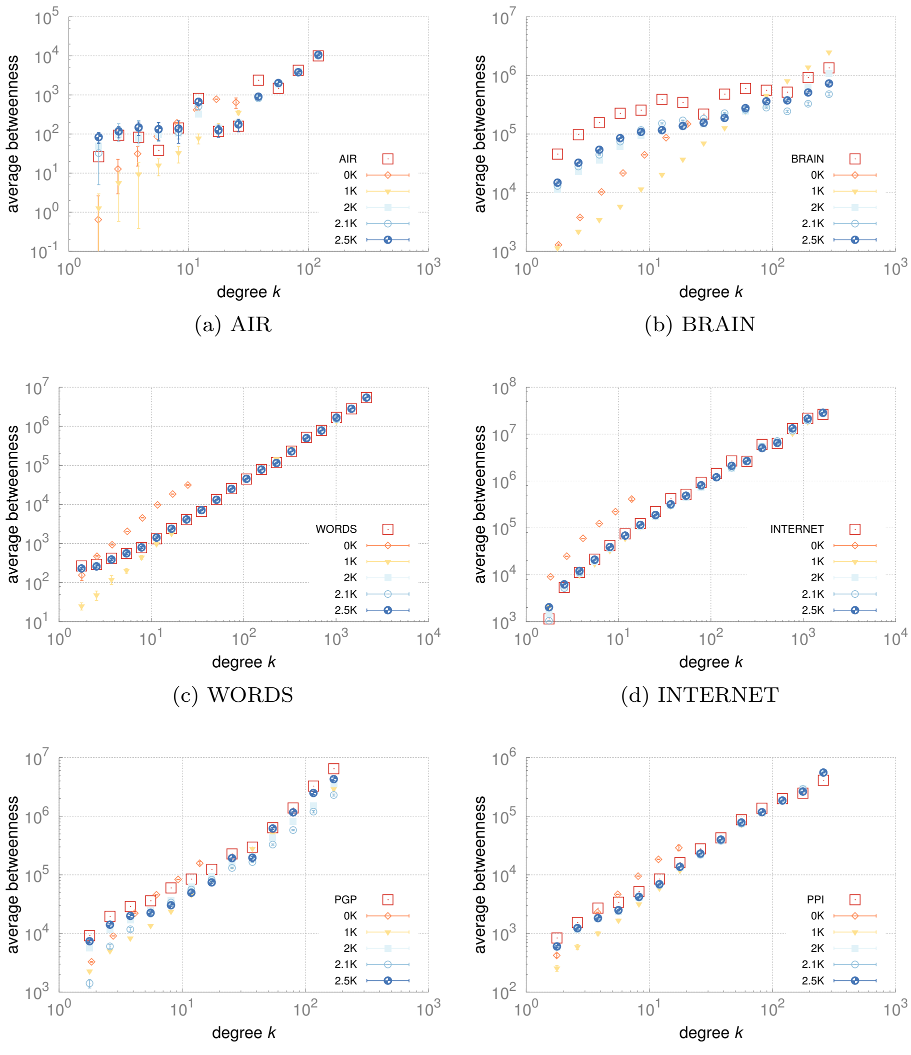

(a) AIR (b) BRAIN

(c) WORDS (d) INTERNET

(e) PGP (f) PPI

Supplementary Figure 8: **Average betweenness of nodes of a given degree in real networks and their** ***dk*****-randomizations.**

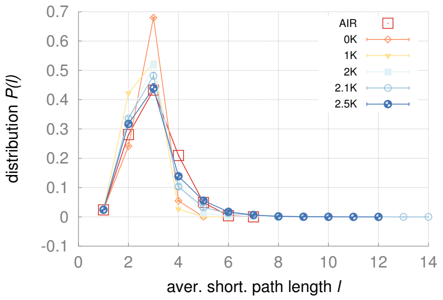

(a) AIR

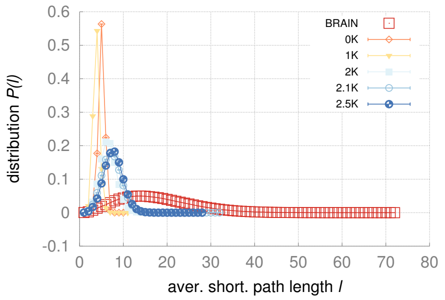

(b) BRAIN

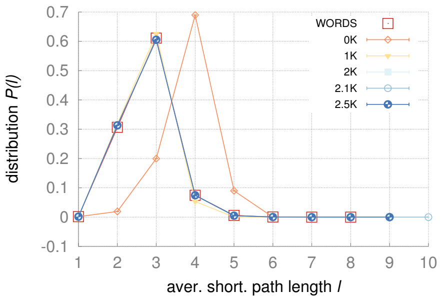

(c) WORDS

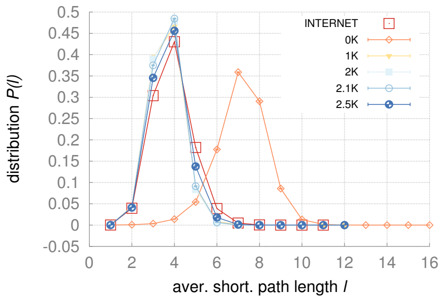

(d) INTERNET

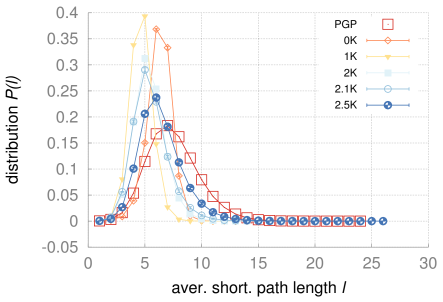

(e) PGP

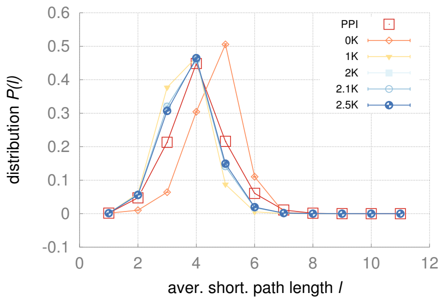

(f) PPI

Supplementary Figure 9: **Shortest path distance distributions in real networks and their dk-randomizations.**

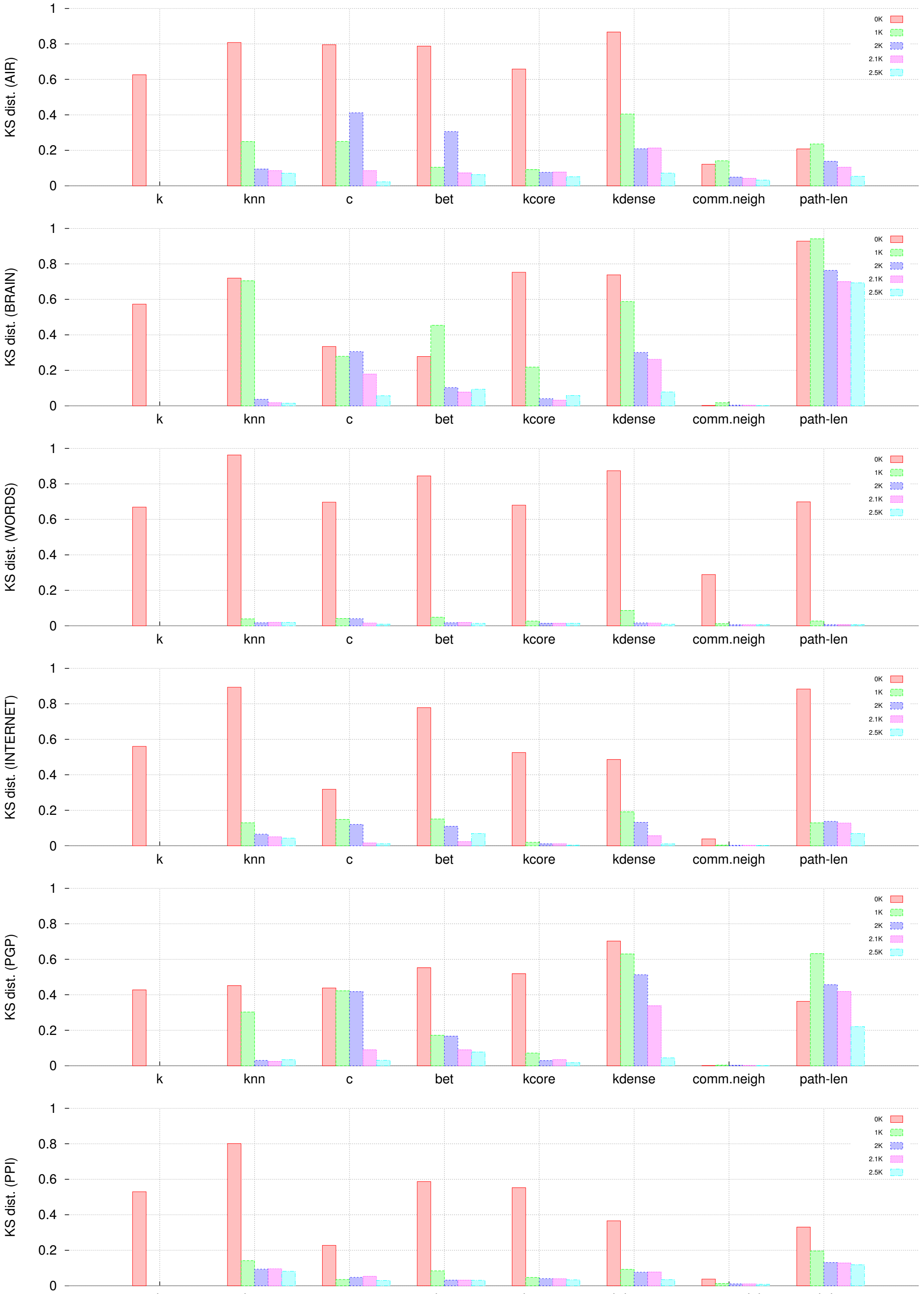

Supplementary Figure 10: **Kolmogorov-Smirnov distances between real networks and their** ***dk*****-randomizations.**

Supplementary Tables

Supplementary Table 1: **Largest eigenvalues, averaged across different realizations for each d, and their standard deviations in parentheses.**

	Original	$0k$	$1k$	$2k$	$2.1k$	$2.5k$
AIR	48.07	12.97(0.08)	42.41(0.24)	47.46(0.01)	47.51(0.02)	47.82(0.03)
BRAIN	119.66	8.91(0.01)	54.89(0.26)	113.41(0.02)	114.09(0.06)	122.27(0.20)
WORDS	109.44	13.06(0.02)	104.12(0.28)	108.82(0.03)	108.80(0.04)	108.92(0.02)
INTERNET	67.17	5.36(0.01)	56.02(0.33)	61.15(0.03)	61.32(0.06)	65.34(0.10)
PGP	42.44	5.77(0.02)	19.50(0.24)	34.08(0.03)	34.40(0.05)	42.95(0.12)
PPI	38.56	8.05(0.05)	32.47(0.17)	34.07(0.04)	34.05(0.04)	35.56(0.10)

Supplementary Table 2: **Spectral gaps, averaged across different realizations for each d, and their standard deviations in parentheses.**

	Original	$0K$	$1K$	$2K$	$2.1K$	$2.5K$
AIR	29.34	6.04(0.09)	32.61(0.46)	37.86(0.25)	37.21(0.21)	30.93(0.29)
BRAIN	40.97	2.90(0.06)	35.52(0.31)	77.53(0.11)	76.59(0.27)	42.71(0.35)
WORDS	65.31	5.86(0.02)	65.28(0.51)	68.53(0.14)	68.47(0.12)	68.21(0.15)
INTERNET	17.56	0.70(0.05)	14.94(0.53)	18.83(0.07)	18.55(0.11)	19.53(0.25)
PGP	4.25	0.98(0.04)	5.51(0.31)	18.01(0.18)	17.55(0.21)	4.71(0.19)
PPI	11.69	2.25(0.07)	15.75(0.27)	16.44(0.19)	16.28(0.20)	10.76(0.17)

Supplementary Table 3: **The considered networks, their abbreviations, and the numbers of nodes and links in them.**

Network	Abbr.	N	M
US air transportation network [1]	AIR	500	2,980
Brain network [2]	BRAIN	17,455	67,895
English word network [3]	WORDS	7,377	44,205
Internet AS-level [4]	INTERNET	20,906	42,994
PGP web of trust [5]	PGP	10,680	24,316
Protein interaction network [6]	PPI	4,099	13,355

Supplementary Table 4: **Parameters used for the dk-randomization (left) and $2.1k/2.5k$-targeting $2k$-preserving (right) rewiring processes (M the number of edges in the real network, $\bar{c}$ average clustering, $\bar{c}(k)$ average clustering of nodes of degree k).**

G_T	dk-randomization		p-targeting dk-preserving rewiring					
	R	d	p_{G_T}	d	R	β_0	β_{factor}	α
AIR	$100M$	1/2	$\bar{c}$, $\bar{c}(k)$	2	$10M$	10^{-2}	1.4	$5 \cdot 10^{-4}$
BRAIN	$100M$	1/2	$\bar{c}$, $\bar{c}(k)$	2	$10M$	10^{-2}	1.1	$5 \cdot 10^{-5}$
WORDS	$100M$	1/2	$\bar{c}$, $\bar{c}(k)$	2	$10M$	10^{-2}	1.4	$5 \cdot 10^{-4}$
INTERNET	$100M$	1/2	$\bar{c}$, $\bar{c}(k)$	2	$10M$	10^{-2}	1.4	$5 \cdot 10^{-4}$
PGP	$100M$	1/2	$\bar{c}$, $\bar{c}(k)$	2	$100M$	10^{-2}	1.1	$5 \cdot 10^{-7}$
PPI	$100M$	1/2	$\bar{c}$, $\bar{c}(k)$	2	$200M$	10^{-2}	1.1	$5 \cdot 10^{-7}$

Supplementary Table 5: **dk-series vs. d-series**

d	dk-statistics	d-statistics
0	k	-
1	$N(k)$	N
2	$N(k, k')$	M
3	$N_\wedge(k, k', k'')$<br>$N_\triangle(k, k', k'')$	W<br>T

Supplementary Notes

Supplementary Note 1: Network properties

Here we describe all the network properties measured and discussed in Supplementary Note 3 and, where meaningful, their relations to dk-series.

1.1 Degree distribution

The distribution $P(k)$ of node degrees k, i.e., the $1k$-distribution, is:

$$P(k) = \frac{N(k)}{N}, \tag{1}$$

where $N(k)$ is the number of nodes of degree k in the network, and N is the total number of nodes in it, so that $P(k)$ is normalized, $\sum_k P(k) = 1$. The $1k$-distribution fully defines the $0k$-distribution, i.e., the average degree $\bar{k}$ in the network, by

$$\bar{k} = \sum_k kP(k), \tag{2}$$

but not *vice versa*.

1.2 Average nearest neighbor degree (ANND)

The average degree $\bar{k}_{nn}(k)$ of nearest neighbors of nodes of degree k is a commonly used projection of the joint degree distribution (JDD) $P(k, k')$, i.e., the $2k$-distribution. The JDD is defined as

$$P(k, k') = \mu(k, k')\frac{N(k, k')}{2M}, \tag{3}$$

where $N(k, k') = N(k', k)$ is the number of links between nodes of degrees k and k' in the network, M is the total number of links in it, and

$$\mu(k, k') = \begin{cases} 2 & \text{if } k = k', \\ 1 & \text{otherwise,} \end{cases} \tag{4}$$

so that $P(k, k')$ is normalized, $\sum_{k,k'} P(k, k') = 1$. The $2k$-distribution fully defines the $1k$-distribution by

$$P(k) = \frac{\bar{k}}{k} \sum_{k'} P(k, k'), \tag{5}$$

but not *vice versa*. The average neighbor degree $\bar{k}_{nn}(k)$ is a projection of the $2k$-distribution $P(k, k')$ via

$$\bar{k}_{nn}(k) = \frac{\bar{k}}{kP(k)} \sum_{k'} k'P(k, k') = \frac{\sum_{k'} k'P(k, k')}{\sum_{k'} P(k, k')}. \tag{6}$$

1.3 Clustering

Clustering of node i is the number of triangles $\triangle_i$ it belongs to, or equivalently the number of links among its neighbors, divided by the maximum such number, which is $k(k-1)/2$, where k is i's degree, $\deg(i) = k$. The average clustering coefficient of the network is

$$\bar{c} = \frac{1}{N} \sum_i \frac{\triangle_i}{k_i(k_i-1)/2} \tag{7}$$

Averaging over all nodes of degree k, the degree-dependent clustering is

$$\bar{c}(k) = \frac{2\triangle(k)}{k(k-1)N(k)}, \text{ where } \triangle(k) = \sum_{i:\,\deg(i)=k} \triangle_i. \tag{8}$$

The degree-dependent clustering is a commonly used projection of the $3k$-distribution. (See [7, 8] for an alternative formalism involving three point correlations.) The $3k$-distribution is actually two distributions characterizing the concentrations of the two non-isomorphic degree-labeled subgraphs of size 3, wedges and triangles:

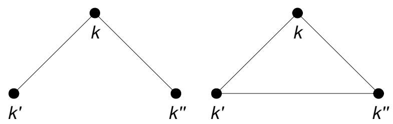

.

Let $N_\wedge(k', k, k'') = N_\wedge(k'', k, k')$ be the number wedges involving nodes of degrees k, k', and k'', where k is the central node degree, and let $N_\triangle(k, k', k'')$ be the number of triangles consisting of nodes of degrees k, k', and k'', where $N_\triangle(k, k', k'')$ is assumed to be symmetric with respect to all permutations of its arguments. Then the two components of the $3K$-distribution are

$$P_\wedge(k', k, k'') = \mu(k', k'') \frac{N_\wedge(k', k, k'')}{2W}, \tag{9}$$

$$P_\triangle(k, k', k'') = \nu(k, k', k'') \frac{N_\triangle(k, k', k'')}{6T}, \tag{10}$$

where W and T are the total numbers of wedges and triangles in the network, and

$$\nu(k, k', k'') = \begin{cases} 6 & \text{if } k = k' = k'', \\ 1 & \text{if } k \neq k' \neq k'', \\ 2 & \text{otherwise,} \end{cases} \tag{11}$$

so that both $P_\wedge(k', k, k'')$ and $P_\triangle(k, k', k'')$ are normalized, $\sum_{k,k',k''} P_\wedge(k', k, k'') = \sum_{k,k',k''} P_\triangle(k, k', k'') = 1$. The $3k$-distribution defines the $2k$-distribution (but not *vice versa*), by

$$P(k, k') = \frac{1}{k + k' - 2} \sum_{k''} \left\{ \frac{6T}{M} P_\triangle(k, k', k'') + \frac{W}{M} \left[P_\wedge(k', k, k'') + P_\wedge(k, k', k'') \right] \right\}. \tag{12}$$

The normalization of $2k$- and $3k$-distributions implies the following identity between the numbers of triangles, wedges, edges, nodes, and the second moment of the degree distribution $\bar{k^2} = \sum_k k^2 P(k)$:

$$2\frac{3T + W + M}{N} = \bar{k^2}. \tag{13}$$

The degree-dependent clustering coefficient $\bar{c}(k)$ is the following projection of the $3k$-distribution

$$\bar{c}(k) = \frac{6T}{N} \frac{\sum_{k',k''} P_{\triangle}(k, k', k'')}{k(k-1)P(k)}. \tag{14}$$

1.4 Subgraph frequencies

The concentration of subgraphs of size 3 is exactly fixed only by the $3k$-distribution, or by the 3-distribution, Supplementary Note 4. There are two non-isomorphic connected graphs of size 3 (triangles and wedges), and their concentrations are defined as

$$C_{\wedge} = \frac{\wedge}{N_3}, \qquad C_{\triangle} = \frac{\triangle}{N_3}, \tag{15}$$

where $\wedge$ is the number of wedges in the graph, $\triangle$ is the number of triangles in the graph, and $N_3 = \wedge + \triangle$ is the total number of connected subgraphs of size 3 in the graph.

The concentration of subgraphs of size 4 is exactly fixed only by the $4k$-distribution, or by the 4-distribution. There are six non-isomorphic connected graphs of size 4,

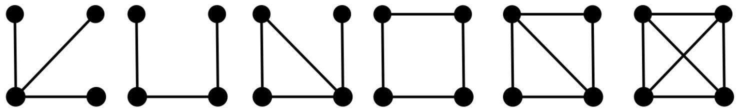

.

and their concentrations are defined as the number of subgraphs of a particular type divided by the total number of connected subgraphs of size 4.

In our comparisons of real networks and their dk-randomizations in Supplementary Note 3 we choose to compare the subgraph concentrations directly, versus computing z-scores, as common in the motif literature. The reasons for this decision is that z-scores are tailored for a fixed null model, while we are considered a series of null models parameterized by d in dk-series. There is nothing in the z-score and dk-series definitions that could easily provide any estimates of how fast the subgraph frequency means and standard deviations in the z-score definition converge as functions of d. Therefore the comparisons of z-scores for different values of d would be meaningless.

1.5 Common neighbors

The number m_{ij} of common neighbors between two connected nodes i and j is the number of nodes to which both i and j are connected, or equivalently the

multiplicity of edge (i, j):

$$m_{ij} = \sum_l A_{il} A_{lj} A_{ij}, \tag{16}$$

where $\{A_{ij}\}$ is the adjacency matrix of the graph. The distribution $P(m)$ of the number of common neighbors m is then

$$P(m) = \frac{\sum_{i<j} \delta_{m_{ij},m}}{N(N-1)/2}, \tag{17}$$

where δ is the Kronecker delta. The common neighbor distribution is thus the probability that two connected nodes in the graph have m common neighbors. This property is exactly fixed only by the $3k$-distribution.

1.6 k-coreness and k-denseness

The k-core decomposition [9] of a graph is a set of nested subgraphs induced by nodes of the same k-coreness. A node has k-coreness equal to k if it belongs to a maximal connected subgraph of the original graph, in which all nodes have degree at least k, i.e., in which each node is connected to at least k other nodes in the subgraph.

Similarly, the k-dense decomposition [10] of a graph is a set of nested subgraphs induced by edges of the same k-denseness. An edge has k-denseness equal to k if it belongs to a maximal connected subgraph of the original graph, in which all edges have multiplicity [7, 8, 11] at least $k-1$, i.e., in which each pair of connected nodes has at least $k-1$ common neighbors in the subgraph.

Both the k-core and k-dense decompositions rely on the analysis of local properties of nodes and edges. However, due to the recursive nature of these decompositions, the dk-distributions with $d = 0, 1, 2, 2.1, 2.5$ do not exactly fix either the k-core or k-dense distributions.

1.7 Betweenness

Betweenness $b(i)$ of node i is a measure of how "important" i is in terms of the number of shortest paths passing through it. Formally, if $\sigma_{st}(i)$ is the number of shortest paths between nodes $s \neq i$ and $t \neq i$ that pass through i, and σ_{st} is the total number of shortest paths between the two nodes $s \neq t$, then betweenness of i is

$$b(i) = \sum_{s,t} \frac{\sigma_{s,t}(i)}{\sigma_{s,t}}. \tag{18}$$

Averaging over all nodes of degree k, degree-dependent betweenness $\bar{b}(k)$ is

$$\bar{b}(k) = \sum_{i:\, \deg(i)=k} \frac{b(i)}{N(k)}. \tag{19}$$

1.8 Shortest path distance

The distance distribution is the distribution of hop-lengths of shortest path between nodes in a network. Formally, if $N(h)$ is the number of node pairs located at hop distance h from each other, then the distance distribution $P(h)$ is

$$P(h) = \frac{N(h)}{N(N-1)/2}, \tag{20}$$

where $N(N-1)/2$ is the total number of nodes pairs in the network. The average distance is:

$$\bar{h} = \sum_h P(h). \tag{21}$$

Finally, the network diameter, i.e., the maximum hop distance between nodes in the network, is

$$d = \max(h). \tag{22}$$

1.9 Spectral properties

The adjacency matrix of graph A gives the full information on the structure of the graph. The largest eigenvalue of A and the spectral gap, which is defined as the difference between the largest and second largest eigenvalue A, play important roles in the dynamic processes on networks. For instance, the largest eigenvalue of the adjacency matrix is related to the speed of the spreading processes on the network [12, 13], while the gap determines the speed of convergence of the random walk to its steady state [14].
The Laplacian matrix describes the diffusion of a random walker on the network and is defined as $L = D - A$, where D is the diagonal matrix of degrees $D_{ij} = \delta_{ij} k_i$, δ_{ij} is Kronecker delta and k_i is the degree of node i. The smallest eigenvalue of the Laplacian matrix is associated to stationary distribution of random walker and it is always equal to zero, while the smallest non-zero eigenvalue, Fiedler value, defines the time scale of the slowest mode of the diffusion [14].

Supplementary Note 2: Considered networks

We apply the *dk*-series analysis to the following six social, biological, language, communication, and transportation networks, Table 3:

- AIR. The US air transportation network [1]. The nodes are airports, and there is a link between two airports if there is a direct flight between them.

- BRAIN. The largest connected component of an fMRI map of the human brain [2]. The nodes are voxels (small areas of a resting brain of approximately 36mm^3 volume each), and two voxels are connected if the correlation coefficient of the fMRI activity of the voxels exceed 0.7.

- WORDS. The largest connected component of the network of adjacent words in Charles Darwin's "The Origin of Species" [3]. The nodes are words, and two words are connected if they are adjacent in the text.

- INTERNET. The topology of the Internet at the level of Autonomous Systems (ASes) [4]. The nodes are ASs (organizations owing parts of the Internet infrastructure), and there is a link between two ASs if they have a business relationship to exchange Internet traffic.

- PGP (considered in the main text). The largest strongly connected component of the technosocial web of trust relationships among people extracted from the Pretty Good Privacy (PGP) data [5]. The nodes are PGP certificates of users, and there is a link between two certificates if their users mutually trust each other's certificate/user associations.

- PPI. The largest connected component of the human protein interaction network [6]. The nodes are proteins, and there is a link between two proteins if they interact.

Table 4 reports the parameters used for each network in the dk-randomization and p-targeting dk-preserving rewiring processes.

Supplementary Note 3: Results

Degree distribution. We observe in Fig. 1 that while $0k$-randomizations are way off, the dk-random graphs with $d \geq 1$ reproduce the degree distributions in the real networks exactly, which is by definition: the $1k$-distribution is the degree distribution, and dk-random graphs with $d \geq 1$ have exactly the same degree distributions as the real networks.

Average nearest neighbor degree (ANND). We observe in Fig. 2 that while $0k$-randomizations are way off, the $1k$-random graphs tend to be closer to the real networks in terms of ANND, whereas the dk-random graphs with $d \geq 2$ have exactly the same average neighbor degrees as the real networks, which is again by definition: the dk-random graphs with $d \geq 2$ have exactly the same JDD $P(k, k')$ as the real networks. In the WORDS, INTERNET, and PPI cases, the ANNDs $\bar{k}_{nn}(k)$ even in the $1k$-random graphs do not noticeably differ from the ANNDs in the real networks.

Clustering. We observe in Fig. 3 that degree-dependent average clustering in the $2.5k$-random graphs matches the one in the real networks, which is again by definition. For $d < 2.5$, degree-dependent clustering differs sensibly in many cases. However, degree-dependent clustering in the AIR network does not exhibit noticeable differences with its $2.1k$-randomizations, while in the WORDS case, even the $1k$-random graphs reproduce degree-depended clustering nearly exactly.

Subgraph frequencies. We observe in Fig. 4 that the $2k$-random graphs reproduce the subgraphs frequencies in most cases, but the BRAIN and PGP require $d = 2.5$ to reproduce these frequencies.

Common neighbors. We observe in Fig. 5 that the $1k$-random graphs reproduce the common neighbor distributions in all the cases except the BRAIN, which requires $d = 2$, and PGP, which requires $d = 2.5$.

k-coreness and k-denseness. We observe in Fig. 6 that the $2k$-random graphs reproduce the k-coreness distributions in all the networks except the PGP and BRAIN that require $d = 2.5$. We observe in Fig. 7 that the $2.5k$-random graphs reproduce the k-denseness distributions in all the networks. The k-denseness distributions in the AIR and WORDS networks are reproduced even by their $2k$-random graphs.

Betweenness. We observe in Fig. 8 that betweenness in the BRAIN network cannot be approximated even by its $2.5k$-random graphs. The INTERNET lies at the other extreme: even the $1k$-random graphs reproduce its betweenness. The PGP network requires all the constraints imposed by the $2.5k$-distribution, while betweenness in all the other networks is similar to betweenness in their $2k$-random graphs.

Shortest path distance. We observe in Fig. 9 that the distance distributions in the INTERNET and WORDS networks are correctly reproduced by their $1k$-random graphs. Even $d = 2.5$ is not enough for the BRAIN, while the same value of $d = 2.5$ suffices for all the networks.

Spectral properties. We observe in Table 1 that the largest eigenvalue of the adjacency matrix is closely, although not exactly, reproduced by $d = 2.5k$-random graphs for all six networks. Furthermore, we observe that the largest eigenvalues for $2k$-random graphs of AIR and WORDS networks are very close to the eigenvalues of the original networks.
The values of the spectral gaps for $2.5k$-random graphs shown in Table 2 are relatively close to the values observed for the original networks, with relative difference for AIR, BRAIN and WORDS networks around 5%. The large values of the spectral gaps for $2k$ and $2.1k$-random graphs indicate that they are more robust, in the sense of being better connected and interlinked, compared to the original networks.

Kolmogorov-Smirnov distance. In Fig. 10 we quantify the convergence of dk-series in terms of Kolmogorov-Smirnov (KS) distances between the distributions of per-node values of a given property in the real networks and the same distributions in their dk-random graphs. We report the KS distances for the following properties:

k degree, cf. Fig. 1;

knn ANND, cf. Fig. 2;

c clustering, cf. Fig. 3;

comm.neigh common neighbors, cf. Fig. 5;

kcore k-coreness, cf. Fig. 6;

kdense k-density, cf. Fig. 7;

bet betweenness, cf. Fig. 8;

path-len shortest path distance, cf. Fig. 9.

The Kolmogorov-Smirnov distance between two cumulative distribution functions (CDFs) $F_1(x)$ and $F_2(x)$ is

$$D = \sup_x |F_1(x) - F_2(x)|. \tag{23}$$

In our case, $F_1(x)$ is the per-node CDF of a given property in a real network, and $F_2(x)$ is the per-node CDF for the same property computed across all different dk-random graph realizations for the network with a given d. We note that the KS distances provides more detailed statistics than the dk-distributions, because the latter do not differentiate between nodes of the same degree, while the former do. For example, even if the $2k$-distributions and consequently ANNDs $\bar{k}_{nn}(k)$ in two different networks are exactly the same, the distributions of average degrees $\bar{k}_{i,nn}$ of neighbors of each individual node i, $i = 1, \ldots, N$, are in general different, so that the KS distance between the two per-node ANND CDFs is in general greater than zero.

Supplementary Discussion

We compare dk-series with the series based on subgraph frequencies, and show that the latter cannot form a systematic basis for topology analysis.

The difference between dk-series and subgraph-based-series, which we can call d-series, is that the former is the series of distributions of d-sized subgraphs labeled with node degrees in a given network, while the d-series is the distributions of such subgraphs in which this degree information is ignored. This difference explains the mnemonic names for these two series: 'd' in 'dk' refers to the subgraph size, while 'k' signifies that they are labeled by node degrees—'k' is a standard notation for node degrees.

This difference between the dk-series and d-series is crucial. The dk-series are inclusive, in the sense that the $(d+1)k$-distribution contains the full information about the dk-distribution, plus some additional information, which is not true for d-series.

To see this, let us consider the first few elements of both series in Table 5. In Supplementary Note 1 we show explicitly how the $(d+1)k$-distributions define the dk-distribution for $d = 0, 1, 2$. The key observation is that the d-series does not have this property. The 0'th element of d-series is undefined. For $d = 1$ we have the number of subgraphs of size 1, which is just N, the number of nodes in the network. For $d = 2$, the corresponding statistics is M, the number of links, subgraphs of size 2. Clearly, M and N are independent statistics, and the former does not define the latter. For $d = 3$, the statistics are W and T, the total number of wedges and triangles, subgraphs of size 3, in the network. These do not define the previous element M either. Indeed, consider the following two networks of size N—the chain and the star:

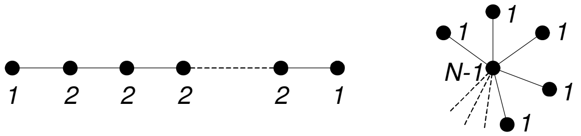

There are no triangles in either network, $T = 0$. In the chain network, the number of wedges is $W = N - 2$, and in the star $W = (N-1)(N-2)/2$. We see that even though W ($d = 3$) scales completely differently with N in the two networks, the number of edges $M = N - 1$ ($d = 2$) is the same.

In summary, d-series is not inclusive. For each d, the corresponding element of the series reflects a differen kind of statistical information about the network topology, unrelated or only loosely related to the information conveyed by the preceding elements. At the same time, similar to dk-series, the d-series is also converging since at $d = N$ it specifies the whole network topology. However, this convergence is much slower that in the dk-series case. In the two networks considered above, for example, neither $W = N - 2,\ T = 0$ nor $W = (N - 1)(N-2)/2,\ T = 0$, fix the network topology as there are many non-isomorphic graphs with the same (W, T) counts, whereas the $3k$-distributions $N_\wedge(1, 2, 2) = 2,\ N_\wedge(2, 2, 2) = N - 4$ and $N_\wedge(1, N-1, 1) = (N-1)(N-2)/2$ define the chain and star topologies exactly.

The node degrees thus provide necessary information about subgraph locations in the original network, which significantly speeds up convergence as a function of d, and more importantly makes the dk-series basis inclusive and systematic.

Supplementary Methods

The methods that we use to sample dk-random graphs for a given graph representing a real network are based on two different rewiring processes: dk-randomizing rewiring ($d = 0, 1, 2$) and p-targeting dk-preserving rewiring ($p = 2.1k, 2.5k$).

The first method (***dk*-randomization**) consists of swapping random pairs of edges in the original network preserving its dk-distribution, Algorithm 1. The following three input parameters are required: G_T the original graph, R the

number of rewirings to apply, and d index that indicates the dk-distribution to preserve. The random edge selection function on line 4 and the rewiring function on line 5 depend on d as follows:

- if $d = 0$, random edge (i, j) and non-edge (a, b) (disconnected nodes a and b) are selected, and the rewiring consists of removing edge (i, j) and adding edge (a, b).
- if $d = 1$, two random edges (i, j) and (a, b) are selected and discarded if either edge (i, b) or edge (j, a) exists; if neither edge (i, b) nor edge (j, a) exists, the rewiring consists of removing edges (i, j) and (a, b), and adding edges (i, b) and (j, a).
- if $d = 2$, two random edges (i, j) and (a, b) such that degrees $k_i = k_a$ are selected and discarded if either edge (i, b) or edge (j, a) exists; if neither edge (i, b) nor edge (j, a) exists, the associated rewiring consists of removing edges (i, j) and (a, b) and adding edges (i, b) and (j, a).

Supplementary Algorithm 1: dk-randomization process.

Input: G_T; // Original graph
Input: R; // Number of rewirings
Input: d; // d value: 0, 1, or 2
/* 1. Initialize process variables */
1 `i = 0 ;` // Rewiring counter
2 G_0 = G_T; // Graph to rewire
/* 2. Apply R dk-rewirings */
3 **while** $i < R$ **do**
    /* Select a random pair of edges (see the text) */
4   `rew = random(edges` $\in G_i$`);`
    /* Apply the rewiring to G_i */
5   `apply_rewiring(`G_i`, rew);`
6     $i = i + 1$;
Output: G_i; // dk-randomized graph

The second method of (p**-targeting** dk**-preserving rewiring**) is based on simulated annealing, and consists of two phases: randomization and targeting rewiring, Algorithm 2. The following input parameters are required: G_T the original graph, p_{G_T} the property to target, R the number of dk-rewirings to apply at each value of temperature, β_0 the initial inverse temperature, β_{factor} the rate of temperature decrease, and α the acceptance threshold. In the first phase the original graph is $2k$-randomized by Algorithm 1. In the second phase, the obtained $2k$-random graph is $2k$-rewired, but each rewiring is accepted with probability $\min[\exp(-\beta H), 1]$ which depends on current values of energy H and temperature $1/\beta$. Energy is defined as the distance between the values of property p in the original and current rewired graphs. Temperature is high initially, but each round of R rewirings (line 9), it decreases by factor β_{factor}, thus decreasing the probability of accepting a rewiring that increases energy. This

second phase terminates when either energy is zero, meaning that the value of p-property in the rewired graph p_{G_i} is equal to its value in the original graph p_{G_T}, or when the percentage of accepted rewirings during the last round falls below a user-specified threshold α. Function `compute_property(`G`)` appearing on lines 3 and 12 returns average clustering $\bar{c}$ or average degree-dependent clustering $\bar{c}(k)$ of G depending on whether $d = 2.1$ or $d = 2.5$, respectively. Energy function `distance(`p_{G_i}, p_{G_T}`)` appearing on lines 4 and 13 depends on d as follows:

- if $d = 2.1$, `distance(`p_{G_i}, p_{G_T}`)` $= |\bar{c}_{G_i} - \bar{c}_{G_T}|$,
- if $d = 2.5$, `distance(`p_{G_i}, p_{G_T}`)` $= \sum_k |\bar{c}_{G_i}(k) - \bar{c}_{G_T}(k)|$.

Code availability We release the software package that implements the dk-randomization algorithms described above. The code is freely available at `http://polcolomer.github.io/RandNetGen/`[15].

Supplementary Algorithm 2: p-targeting dk-preserving rewiring process.

Input: G_T; // Original graph
Input: R; // Number of rewirings
Input: p_{G_T}; // Target value of the property
Input: β_0; // Initial inverse temperature
Input: β_{factor}; // Temperature decrease rate
Input: α; // Acceptance threshold

/* 1. $2k$-randomize the original graph using Alg. 1 */
M = num.edges(G_T);
G_0 = dk-randomize(G_T, $R = 100M$, $d = 2$);
/* 2. Compute the current value of the property */
p_{G_0} = compute_property(G_0);
/* 3. Compute the current energy, the distance between p_{G_0} and p_{G_T} */
H_0 = distance(p_{G_0}, p_{G_T});
/* 4. Initialize process variables */
i = 0; // Accepted rewirings counter
step = 0; // Counter associated with current temperature
β_{step} = β_0;
A_{step} = 1; // Acceptance ratio, percentage of accepted rewirings that modified energy at current temperature
/* 5. $2k$-rewire until energy goes to zero or the acceptance ratio falls below the threshold */
while $H_i > 0$ **and** $A_{step} > \alpha$ **do**
  /* Apply R p-targeting $2k$-rewirings at constant temperature $1/\beta_{step}$ */
  for $r \leftarrow 1$ **to** R **do**
    /* Apply a single $2k$-rewiring to the current graph, using Alg. 1 */
    G_{hyp} = dk-randomize(G_i, 1, 2);
    p_{hyp} = compute_property(G_{hyp});
    /* Compute current energy */
    H_{hyp}= distance(p_{hyp}, p_{G_T});
    /* Compute energy change */
    $\Delta H = H_{hyp} - H_i$;
    /* Accept graph G_{hyp} with the following probability */
    with probability $\min(e^{-\beta_{step}\cdot\Delta H}, 1)$ **do:**
      $r = r + 1$;
      $i = i + 1$;
      $G_i = G_{hyp}$;
      $H_i = H_{hyp}$;
  $\beta_{step+1} = \beta_{step} \cdot \beta_{factor}$; // Update β
  $A_{step+1} = \frac{\#\text{rewirings accepted with } \Delta H \neq 0}{\#\text{rewirings with } \Delta H \neq 0}$; // Update the acceptance ratio
  step = step + 1;
Output: G_i

Supplementary References

[1] Vittoria Colizza, Romualdo Pastor-Satorras, and Alessandro Vespignani. Reaction-diffusion Processes and Metapopulation Models in Heterogeneous Networks. *Nat Phys*, 3:276–282, 2007. doi:10.1038/nphys560.

[2] Victor Eguíluz, Dante Chialvo, Guillermo Cecchi, Marwan Baliki, and A. Vania Apkarian. Scale-Free Brain Functional Networks. *Phys Rev Lett*, 94(1):018102, January 2005. doi:10.1103/PhysRevLett.94.018102.

[3] R Milo, S Itzkovic, N Kashtan, R Levitt, S Shen-Orr, I Ayzenshtat, M Sheffer, and U Alon. Superfamilies of Evolved and Designed Networks. *Science*, 303:1538–1542, 2004. doi:10.1126/science.1089167.

[4] Priya Mahadevan, Dmitri Krioukov, Marina Fomenkov, Bradley Huffaker, Xenofontas Dimitropoulos, Kc Claffy, and Amin Vahdat. The Internet AS-Level Topology: Three Data Sources and One Definitive Metric. *Comput Commun Rev*, 36(1):17–26, 2006. doi:10.1145/1111322.1111328.

[5] Marián Boguñá, Romualdo Pastor-Satorras, Albert Díaz-Guilera, and Alex Arenas. Models of social networks based on social distance attachment. *Phys Rev E*, 70(5):056122, November 2004. doi:10.1103/PhysRevE.70.056122.

[6] Thomas Rolland, Murat Tas, Nidhi Sahni, Song Yi, Irma Lemmens, Celia Fontanillo, Roberto Mosca, Atanas Kamburov, Susan D Ghiassian, Xinping Yang, Lila Ghamsari, Dawit Balcha, Bridget E Begg, Pascal Braun, Marc Brehme, Martin P Broly, Anne-ruxandra Carvunis, Dan Convery-zupan, Roser Corominas, Changyu Fan, Eric Franzosa, Jasmin Coulombe-huntington, Elizabeth Dann, Matija Dreze, Fana Gebreab, Bryan J Gutierrez, Madeleine F Hardy, Mike Jin, Shuli Kang, Ruth Kiros, Guan Ning Lin, Ryan R Murray, Alexandre Palagi, Matthew M Poulin, Katja Luck, Andrew Macwilliams, Xavier Rambout, John Rasla, Patrick Reichert, Viviana Romero, Elien Ruyssinck, Julie M Sahalie, Annemarie Scholz, Akash a Shah, Amitabh Sharma, Yun Shen, Kerstin Spirohn, Stanley Tam, Alexander O Tejeda, Shelly a Trigg, Jean-claude Twizere, Kerwin Vega, and Jennifer Walsh. A Proteome-Scale Map of the Human Interactome Network. *Cell*, 159:1212–1226, 2014. doi:10.1016/j.cell.2014.10.050.

[7] M. Ángeles Serrano and Marián Boguñá. Clustering in complex networks. I. General formalism. *Phys Rev E*, 74(5):056114, November 2006. doi:10.1103/PhysRevE.74.056114.

[8] M. Ángeles Serrano and Marián Boguñá. Clustering in complex networks. II. Percolation properties. *Phys Rev E*, 74(5):056115, August 2006. doi:10.1103/PhysRevE.74.056115.

[9] José Alvarez-Hamelin, Luca Dall'Asta, Alain Barrat, and Alessandro Vespignani. K-core decomposition of Internet graphs: hierarchies, self-similarity and measurement biases. *Networks Heterog Media*, 3(2):371–393, March 2008. doi:10.3934/nhm.2008.3.371.

[10] Kazumi Saito and Takeshi Yamada. Extracting Communities from Complex Networks by the k-dense Method. In *Sixth IEEE Int Conf Data Min - Work*, pages 300–304. IEEE, 2006. doi:10.1109/ICDMW.2006.76.

[11] Vinko Zlatić, Diego Garlaschelli, and Guido Caldarelli. Networks with arbitrary edge multiplicities. *EPL*, 97(2):28005, January 2012. doi:10.1209/0295-5075/97/28005.

[12] Yang Wang, Deepayan Chakrabarti, Chenxi Wang, and Christos Faloutsos. Epidemic spreading in real networks: An eigenvalue viewpoint. In *Proceedings of the 22nd International Symposium on Reliable Distributed Systems*, pages 25–34. IEEE, 2003. doi:10.1109/RELDIS.2003.1238052.

[13] Gregorio D'Agostino, Antonio Scala, Vinko Zlatić, and Guido Caldarelli. Robustness and assortativity for diffusion-like processes in scale-free networks. *EPL*, 97(6):68006, 2012. doi:10.1209/0295-5075/97/68006.

[14] Piet Van Mieghem. *Graph spectra for complex networks*. Cambridge University Press, Cambridge, 2010.

[15] Pol Colomer de Simon. RandNetGen: a Random Network Generator. URL: http://polcolomer.github.io/RandNetGen/.
